# Differential Gene Expression Supports a Resource‐Intensive, Defensive Role for Colony Production in the Bloom‐Forming Haptophyte, *Phaeocystis globosa*

**DOI:** 10.1111/jeu.12727

**Published:** 2019-03-27

**Authors:** Margaret Mars Brisbin, Satoshi Mitarai

**Affiliations:** ^1^ Marine Biophysics Unit Okinawa Institute of Science and Technology Graduate University Onna‐Son Japan

**Keywords:** Algae bloom, algal bloom, colonial morphotype, dimethylsulfide, dimethylsulfoniopropionate, phytoplankton, plankton, RNA‐seq, transcriptome, transcriptomics

## Abstract

*Phaeocystis globosa* forms dense, monospecific blooms in temperate, northern waters. Blooms are usually dominated by the colonial morphotype—nonflagellated cells embedded in a secreted mucilaginous mass. Colonial *Phaeocystis* blooms significantly affect food‐web structure and function and negatively impact fisheries and aquaculture, but factors regulating colony formation remain enigmatic. Destructive *P. globosa* blooms have been reported in tropical and subtropical regions more recently and warm‐water blooms could become more common with continued climate change and coastal eutrophication. We therefore assessed genetic pathways associated with colony formation by investigating differential gene expression between colonial and solitary cells of a warm‐water *P. globosa* strain. Our results illustrate a transcriptional shift in colonial cells with most of the differentially expressed genes downregulated, supporting a reallocation of resources associated with forming and maintaining colonies. Dimethylsulfide and acrylate production and pathogen interaction pathways were upregulated in colonial cells, suggesting a defensive role for producing colonies. We identify several protein kinase signaling pathways that may influence the transition between morphotypes, providing targets for future research into factors affecting colony formation. This study provides novel insights into genetic mechanisms involved in *Phaeocystis* colony formation and provides new evidence supporting a defensive role for *Phaeocystis* colonies.

*PHAEOCYSTIS* is a cosmopolitan bloom‐forming haptophyte genus encompassing six species (Andersen et al. 2015; Schoemann et al. 2005). Most *Phaeocystis* species (*P. globosa, P. antarctica, P. pouchetii*, and *P. jahnii*) exhibit a polymorphic life‐cycle, alternating between colonial and free‐living morphotypes. *Phaeocystis* blooms are usually dominated by the colonial morphotype and are typically very dense, produce large biomasses, and impact food‐web structure and function (Schoemann et al. 2005). *Phaeocystis* is a major contributor to dimethylsulfoniopropionate (DMSP) and dimethylsulfide (DMS) production globally (Liss et al. 1994) and regional peaks in DMS production are closely correlated with colonial *Phaeocystis* blooms (Van Duyl et al. 1998). DMS produced in the surface ocean is aerosolized and its oxidation products promote cloud formation, increase albedo, and affect global climate (Charlson et al. 1987). In algal cells, DMSP and its cleavage products, DMS and acrylate, contribute to osmotic balance, neutralize reactive oxygen species, and deter grazing (Noordkamp et al. 2000; Sunda et al. 2002). Despite the ecological importance of colony formation in *Phaeocystis*, triggers for transitioning to the colonial morphotype remain enigmatic, and the functional role of colony formation in the *Phaeocystis* life‐cycle is not clearly delineated (Peperzak and Gabler‐Schwarz 2012).

Myriad factors have been studied with regard to their roles in influencing colony formation in *Phaeocystis* species, including nutrient and light availability (Bender et al. 2018; Cariou et al. 1994; Wang et al. 2011), temperature (Verity and Medlin 2003), mechanical stress (Cariou et al. 1994), grazing cues (Long et al. 2007; Tang 2003; Wang et al. 2015), and viral infection (Brussaard et al. 2005, 2007). However, these studies used different *Phaeocystis* species, strains, and morphotypes with a range of experimental conditions, which yielded variable and sometimes contradictory results. Nonetheless, several lines of evidence suggest that colony formation serves a defensive role. First, while viruses can cause 30–100% cell lysis in solitary *Phaeocystis,* viruses rarely infect colonial cells, which lyse primarily due to nutrient limitation (Brussaard et al. 2005, 2007). Second, ciliates and other microzooplankton that graze solitary *Phaeocystis* are unable to graze on colonies (Tang et al. 2001) and chemical cues from these grazers induce colony formation and increased colony size (Long et al. 2007; Tang 2003). Third, acrylate, which is produced with DMS when DMSP is cleaved, accumulates within colonies and may further deter macro‐ and micro‐grazers and heterotrophic bacteria (Hamm 2000; Noordkamp et al. 2000). However, while cellular growth rate increases in colonial cells relative to solitary cells when colony formation is induced in nutrient‐rich conditions, growth rate decreases when colonies are induced under nutrient‐limiting conditions (Wang et al. 2015). Thus, colony formation can defend against pathogens and grazers, but it is costly (Wang et al. 2015), suggesting that colony formation is likely a complex response to interacting biotic and abiotic factors (Long et al. 2007).

Colony formation may play a fundamental role in *Phaeocystis* reproduction. *Phaeocystis* has one of the most complex and polymorphic life cycles among phytoplankton genera, and despite extensive study, it remains largely unresolved in most species. Studies have implicated at least six different life stages and up to 15 functional components to the life‐cycle (Gaebler‐Schwarz et al. 2010). In *P. globosa,* four morphotypes are believed to exist: diploid colonial cells devoid of scales and flagella, diploid scale‐free flagellates arising from mechanically disrupted colonies, and two types of small, scaled, haploid flagellates—those that produce vesicles containing star‐shaped alpha‐chitin filaments and those that do not (Rousseau et al. 2007). Haploid flagellates may fuse (syngamy) to produce diploid colony‐forming cells, which in turn undergo meiosis and produce haploid flagellates (Rousseau et al. 2013). Haploid flagellates are often observed swarming inside colonies, suggesting that colonial bloom formation may contribute to successful sexual reproduction in *Phaeocystis* (Peperzak et al. 2000; Rousseau et al. 2013). However, neither syngamy nor meiosis have been directly observed in *Phaeocystis* spp. (Peperzak and Gabler‐Schwarz 2012), even though both events have been documented in several other haptophyte genera (Houdan et al. 2004). If colonial *Phaeocystis* blooms are necessary for sexual reproduction, it would further justify the resource costs associated with colony formation.

Historically, colonial *Phaeocystis* blooms have been restricted to cold, high‐latitude waters—*P. globosa* blooms in the English Channel and North Sea, *P. pouchetii* in the North Atlantic and Arctic, and *P. antarctica* in the Southern Ocean (reviewed in Schoemann et al. 2005). In the last two decades, however, blooms have increasingly been reported in tropical and subtropical regions, including the subtropical N. Atlantic (Long et al. 2007) and the subtropical and tropical South China Sea (Chen et al. 2002; Doan‐Nhu et al. 2010; Liu et al. 2015). Decaying colonial biomass sinks and produces anoxic conditions, making *Phaeocystis* blooms detrimental to benthic fisheries and aquaculture (Desroy and Denis 2004; Peperzak and Poelman 2008; Spilmont et al. 2009). In warmer waters, *Phaeocystis globosa* blooms have been especially catastrophic to local aquaculture (Chen et al. 2002; Doan‐Nhu et al. 2010), possibly because the hemolytic activity of *P. globosa* liposaccharides increases with temperature (Peng et al. 2005). Global climate change and increasing nutrient pollution in coastal regions may mean harmful *Phaeocystis* blooms will continue to increase in range and frequency. Given the ecological impact of colonial *Phaeocystis* blooms and their complex and enigmatic initiating factors, particularly in warm waters, it is imperative to better understand the regulation of colony formation.

Transcriptional approaches have become an exceptionally useful tool to illuminate physiological responses to environmental cues and genes associated with specific life stages in algae and other protists (Caron et al. 2017). In this study, we investigated genetic regulation of colony formation by analyzing gene expression in colonial and flagellated morphotypes of a warm‐water *Phaeocystis globosa* strain. Since *Phaeocystis* is an important marine producer of DMSP and DMS—and because these molecules may be associated with colonial defense, we queried our dataset for algal genes involved in DMSP production (*DSYB*, Curson et al. 2018) and its cleavage to DMS and acrylate (*Alma1*, Alcolombri et al. 2015). *DSYB* and *Alma1* are the only algal genes that encode proteins experimentally proven to catalyze DMSP, DMS, and acrylate production, but neither has been identified in *P. globosa* previously. Overall, our results demonstrate a dramatic transcriptional shift in colonial *P. globosa*, with the vast majority of differentially expressed (DE) genes downregulated in colonial cells. Such a strong transcriptional shift supports an allocation of resources toward colony formation and away from other cellular processes such as translation, cell growth, and cell division. Genes associated with DMSP production (*DSYB*‐like) were not DE, but an *Alma* family‐like gene was upregulated in colonies, suggesting colonies may produce more DMS and acrylate than solitary cells. Colonial cells expressed different pathogen interaction pathways than solitary cells, lending support for colonies serving a defensive role. Additional results implicate several cell‐signaling pathways as important to colony formation. The results from this study provide new insights into the functional role of *Phaeocystis* colonies and physiological processes associated with colony formation. These insights will guide future investigations into factors that influence colony formation in harmful *Phaeocystis* blooms.

## Materials and Methods

### Culture strain and maintenance

Starter cultures of *Phaeocystis globosa* CCMP1528, a warm‐water, colony‐forming *P. globosa* strain (Wang et al. 2011), were purchased from the National Center for Marine Algae and Microbiota (NCMA, East Boothbay, ME) in January 2016. CCMP1528 did not initially form colonies in our culture conditions, but it is known that *P. globosa* strains sometimes stop forming colonies in culture (Janse et al. 1996). We maintained noncolonial cultures in replicate 150 ml Erlenmeyer flasks with 100 ml L1‐Si media in ambient light and temperature on a gently rotating twist mixer (TM‐300, AS ONE, Osaka, Japan, speed setting 1). Culture media was prepared by enriching 34 ppt autoclaved artificial seawater prepared from milliQ water and sea salts (Marine Art SF‐1, AS ONE, Osaka, Japan) with the NCMA L1 media kit (‐Si) and filtering through sterile 0.22 μm pore‐size filters. Cultures were diluted biweekly with freshly prepared media. In the winter of 2017, one *P. globosa* CCMP1528 culture replicate began forming colonies again. It is not clear why this strain resumed forming colonies, but we hypothesize that changes in light intensity and the light:dark cycle during maintenance in ambient light conditions instead of constant light conditions (13:11 at NCMA) affected colony formation. Additionally, artificial seawater media instead of the natural seawater media used at NCMA could have influenced colony formation either directly or by altering the microbial community in the culture. Nonetheless, experimental culture conditions were promptly initiated to investigate gene expression in colonial and solitary cells of the same strain.

### Experimental culture conditions

**Figure 1:**
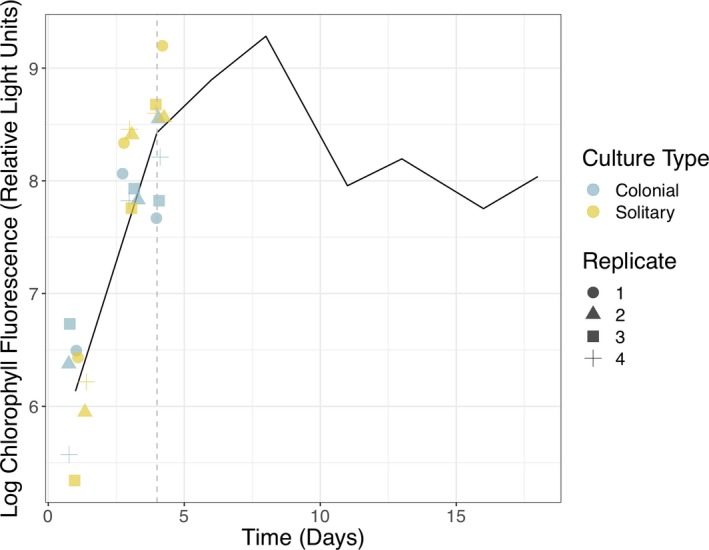
Growth in *Phaeocystis globosa* CCMP1528 culture replicates monitored by in vivo chlorophyll fluorescence. Biological replicates of *P. globosa* CCMP1528 cultures with colonies (blue) and with only solitary cells (yellow), were grown under identical culture conditions. Aliquots were transferred to a microwell plate one, three, and 4 days after culture inoculation and chlorophyll fluorescence was directly measured at ex440 nm and em685 nm with a Tecan Ultra Evolution microplate reader. Fluorescence measurements were compared to a growth curve (black line) prepared prior to this experiment by measuring chlorophyll fluorescence every other day in solitary *P. globosa* CCMP1528 cultures grown in the same culture conditions (mean of *n* = 3). Colonial and solitary replicates were harvested for RNA extraction 4 days after culture inoculation (gray dashed line). Results plotted with R package ggplot2.

We prepared four biological replicates each of colony‐forming and noncolonial *P. globosa* CCMP1528 by inoculating 45 ml of sterile L1 media with 1 ml stock culture in 50‐ml Erlenmeyer flasks. Replicates were placed on a gently rotating twist mixer in a plant growth chamber with cool white fluorescent lamps (CLE‐305, TOMY, Tokyo, Japan) set to 22 °C with light level 4 and a 12:12 day:night ratio. A HOBO temperature and light logger (Onset, Bourne, MA) was kept in the growth chamber during the experiment. The daytime temperature was 21 °C with about 1900–2000 Lux (~30 μmol/m^2^/s) light intensity, and the nighttime temperature was 22 °C. Positions of replicates were rotated daily to prevent position in the chamber from systematically affecting replicates. Four days after initiating experimental conditions, fluorescence was measured for each replicate and 1 ml was transferred to 45 ml of sterile L1 media in a clean 50‐ml flask. The experimental setup was then repeated, allowing for acclimation to the experimental conditions. In the second round, chlorophyll fluorescence was measured 1, 3, and 4 days after inoculation in the middle of the light period. Fluorescence was measured by transferring 200‐μl aliquots from each replicate to wells in a black 96‐well plate (ThermoFisher, Waltham, MA) and recording fluorescence (excitation: 440 nm, emission: 685 nm) with a Tecan Ultra Evolution microplate reader (Tecan, Mannedorf, Switzerland). Growth phase was determined by comparing fluorescence to a growth curve prepared prior to this experiment for solitary *Phaeocystis globosa* CCMP 1528 grown in the same culture conditions. Algal cells were harvested for RNA extraction on day four of the second experimental culture round, when replicates were in the late exponential growth phase (Fig. 1).

**Figure 2:**
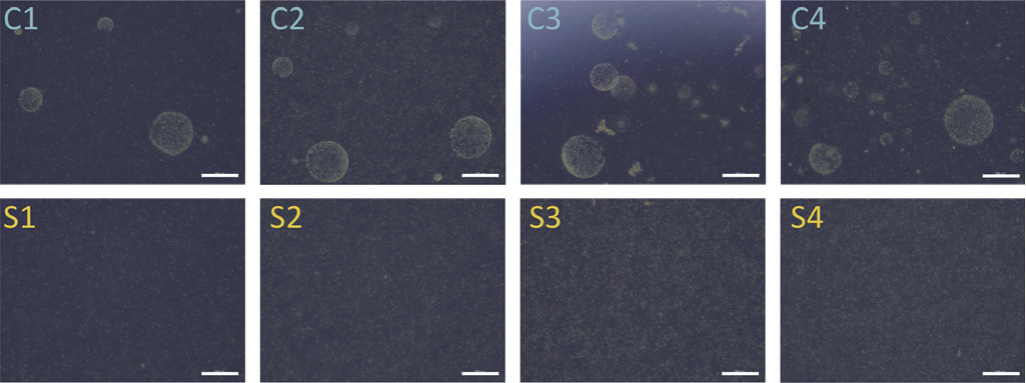
Light micrographs of colonial and solitary culture replicates immediately prior to RNA extraction. Each colonial (**C1**–**C4**) and solitary (**S1**–**S4**) replicate was imaged with an Olympus CKX53 light microscope just before harvesting cells for RNA extraction. Colonies were visible in all colonial replicates and absent in all solitary replicates. Colonial replicate C2 had a high density of solitary cells compared to the other colonial replicates at the time of RNA extraction. Scale bars are 500 μm in all images.

Prior to RNA extraction, each culture replicate was imaged with light microscopy (Olympus CKX53, Waltham, MA) to ensure that colony‐forming replicates were indeed producing colonies and that noncolonial replicates were not (Fig. 2). Colony‐forming culture replicates were filtered through polytetrafluoroethylene (PTFE) filters (10‐μm pore‐size) (Millipore, Burlington, MA) under gentle vacuum. Colonies were visible by eye on the filter surface and swimming flagellates were observed in the flow‐through when viewed with light microscopy, but flagellates were unavoidably retained on the filter as well. The noncolonial culture replicates were first filtered through 50‐μm nylon mesh to remove culture debris, and then filtered through 1.0‐μm pore‐size PTFE filters. No flagellates were visible when the filtrates were viewed with light microscopy. Filters were immediately flash frozen in liquid nitrogen and stored at −80 °C until RNA extraction.

### RNA extraction, library preparation and sequencing

Total RNA was extracted from filters by following the manufacturer's protocols for the MoBio PowerWater RNA extraction kit (Qiagen, Venlo, The Netherlands) including the optional initial heating step. Following extraction, we assessed RNA quality and concentration. RNA extracts were diluted so that 10 ng of RNA were used for each sample with the SMART‐seq v4 Ultra Low Input RNA Kit (Clontech/Takara, Mountain View, CA) and supplemented with 2 μl of a 1:10,000 dilution of External RNA Controls Consortium (ERCC) spike‐in mix one (Ambion, Waltham, MA), an internal quality control. The SMART‐seq kit employs poly‐A priming to target eukaryotic mRNA and to reduce the amount of ribosomal and bacterial RNA present in sequencing libraries. The quality and concentration of the resulting cDNA was assessed before continuing with the manufacturer's protocols for the Nextera XT DNA Library Prep Kit (Illumina, San Diego, CA). Finally, we checked cDNA fragment size before submitting eight cDNA libraries to the Okinawa Institute of Science and Technology DNA Sequencing Section, where the libraries were pooled and sequenced across eight lanes of an Illumina Hiseq4000 flow‐cell to produce paired‐end 150 × 150 bp reads.

### Bioinformatic processing and quality control

Sequencing reads were processed with Trimmomatic software to remove adapter sequences and to filter low‐quality sequences (Bolger et al. 2014). Read quality was checked with FastQC before and after trimming to ensure adapters were removed (Andrews 2010). Remaining reads were mapped to ERCC reference sequences (Cronin et al. 2004) and mapped reads were counted with RSEM software for each sample (Li and Dewey 2011). Counts were further analyzed in the R statistical environment (R Core Team 2013) to assess the relationship between ERCC sequence read counts and their original concentrations in the ERCC standard. Reads mapping to the ERCC reference sequences were then removed from the sequences for each sample with SAMtools (Li et al. 2009) and BEDTools (Quinlan and Hall 2010).

### Transcriptome assembly, assessment, and functional annotation

The Marine Microbial Eukaryote Transcriptome Sequencing Project (MMETSP, Keeling et al. 2014) assembled a transcriptome for *Phaeocystis* sp. CCMP2710, which groups with the *Phaeocystis globosa* species complex in phylogenetic analyses (Fig. S1). Only 25% of our reads, however, mapped to this reference transcriptome. We therefore assembled a de novo transcriptome for *Phaeocystis globosa* CCMP1528 to serve as a reference for read mapping. We used Trinity software for transcriptome assembly (Grabherr et al. 2013) and dereplicated the transcriptome by removing reads with 95% similarity, using CD‐HIT‐EST (Fu et al. 2012). Bacterial sequences were removed by performing a blastn query against the NCBI nucleotide database (downloaded March 2018, ncbi‐blast v2.6.0+, Camacho et al. 2009) and parsing results to identify and remove bacterial contigs. The final assembly was assessed for completeness by searching for eukaryote and protist Benchmarking Universal Single‐Copy Orthologs (BUSCO v3, Simão et al. 2015) within the assembly using the HMMER3 software (Eddy 2011). BUSCOs provide a method to quantitatively assess the quality of a transcriptome in terms of gene content: more complete transcriptomes contain more full‐length BUSCOs, which are well‐conserved genes appearing only once in complete, representative genomes for each BUSCO group. BUSCO completeness scores for the assembly were compared with those for the MMETSP *Phaeocystis* sp. CCMP2710 transcriptome.

We annotated the CCMP1528 transcriptome using two different databases and functional annotation methods: Pfam (Finn et al. 2015) and Kyoto Encyclopedia of Genes and Genomes (KEGG, Kanehisa et al. 2016). Pfam annotation was performed with the dammit software (http://dib-lab.github.io/dammit/), which wraps Transdecoder to translate transcriptome contigs to the longest possible amino acid sequence (Haas et al. 2013), and HMMER to assign Pfam protein homologs to sequences (Eddy 2011). After discarding annotations with *e*‐values greater than 1E‐5, the Pfam annotation with the lowest e‐value was selected for each contig. The Pfam annotations were matched to corresponding Gene Ontology (GO) terms using the Gene Ontology Consortium's Pfam2GO mapping (geneontology.org/external2go/pfam2go, version 07/14/2018, Mitchell et al. 2015). KEGG Orthology (KO) annotation was performed using the GhostKOALA tool with the Transdecoder translated amino acid sequences (kegg.jp/ghostkoala, 05/21/2018, Kanehisa et al. 2016). The KEGG Orthology numbers were then used to access the KEGG API (kegg.jp/kegg/rest/keggapi.html, July 2018) and assign KEGG pathways to transcriptome contigs.

### Differential gene expression analysis

Quality filtered sequences from each of the eight samples were mapped to the assembled *P. globosa* CCMP1528 transcriptome and counted with RSEM software. Counts for each sample were imported into the R statistical environment. Genes without an expression level of at least one FPKM (Fragments Per Kilobase of transcript per Million mapped reads) in at least one sample were removed before differential gene expression testing between colonial and solitary culture replicates was performed with the DESeq function in the Bioconductor package DESeq2 (Love et al. 2014). Genes that were DE with a False Discovery Rate (FDR) adjusted *P*‐value (padj) less than 0.05 were considered statistically significant and included in enrichment testing.

### Gene set enrichment testing

We identified GO terms enriched among significantly upregulated and downregulated genes by applying a hypergeometric test in the R package GOstats (Falcon and Gentleman 2007). GoStats accommodates user‐defined GO annotations, which are necessary when studying nonmodel organisms like *Phaeocystis*. Likewise, a hypergeometric test for significant enrichment of KEGG pathways was applied using the enricher function from the R package ClusterProfiler (Yu et al. 2012). Due to the lower annotation rate, KEGG pathway enrichment was further investigated by additionally applying linear model analysis with the kegga function in the R package edgeR (Robinson et al. 2010). GO terms and KEGG pathways were considered significantly enriched when the statistical test returned a *P*‐value less than 0.05.

### Genes associated with dimethylsulfoniopropionate and dimethylsulfide production

Because *Phaeocystis* is a profusive producer of DMSP and DMS, we specifically queried our dataset for recently discovered algal genes involved in DMSP production (*DSYB*) and its cleavage to DMS and acrylate (*Alma* family genes). We performed blastp queries with curated DSYB protein sequences (provided by Curson et al. 2018) and *E. huxleyi* and *Symbiodinium* Alma family protein sequences downloaded from UniProt (July 2018) against amino acid sequences translated from the *Phaeocystis globosa* CCMP1528 transcriptome. Expression levels of putative *Phaeocystis globosa DSYB* and *Alma* family genes were then checked in colonial and solitary culture replicates.

## Results

### Bioinformatic processing and quality control

Sequencing for this project produced over 1.9 billion read pairs with 159–383 million read pairs per sample. The reads for each sample were deposited in the Sequence Read Archive (SRA) with accession numbers SRR7811979–SRR7811986. Following quality filtering with Trimmomatic, 1.7 billion read pairs remained, with 140–341 million read pairs per sample (Table S1). After mapping reads from each sample to ERCC reference sequences, we plotted log2 FPKM for each sequence against log2 concentration in the standard mix. A simple linear regression was fitted for each sample and *R* ^2^ values ranged from 0.93 to 0.937 (Fig. S2). The strong correlation between observed FPKM and initial concentration for ERCC sequences indicates minimal bias was introduced during PCR amplification, library preparation, and sequencing.

### Transcriptome assembly, assessment, and functional annotation

The final assembly of the *Phaeocystis globosa* CCMP1528 transcriptome included 69,528 contigs and a total of 43.9 Mbp (available for download from https://doi.org/10.5281/zenodo.1476491). The CCMP1528 transcriptome was about three times larger than the MMETSP CCMP2710 transcriptome, but the minimum, maximum, and mean contig lengths were about the same for both (Table S2). When Transrate software was used to align the two transcriptomes, only 18% of CCMP1528 contigs aligned to the CCMP2710 transcriptome, but 55% of the CCMP2710 contigs aligned to the CCMP1528 transcriptome. The *P. globosa* CCMP1528 transcriptome contained more eukaryotes (Complete: 249, Fragmented: 36, Missing: 18) and protist Bench‐marking Universal Single Copy Orthologs (C: 148, F: 3, M: 64) than the MMETSP *Phaeocystis* sp. CCMP2710 transcriptome (Fig. S3). Therefore, the CCMP1528 transcriptome is more complete than the CCMP2710 transcriptome and provides a better reference for differential gene expression testing in this study.

Functional annotation was successful for 26% of the CCMP1528 contigs using HMMER software with the Pfam protein family database, and 50% of the Pfam annotations could be mapped to GO terms for enrichment analysis. Only 14% of contigs was annotated with a KEGG Orthology, but more than half of the KEGG annotated contigs could be assigned to KEGG pathways for enrichment analysis (Table 1). It is common for de novo transcriptomes for marine protists to have low annotation rates (e.g. Kuo et al. 2013; Lauritano et al. 2017; Santoferrara et al. 2014). Additionally, haptophyte transcriptomes will have low annotation rates because haptophytes occupy a phylogenetically unique position on the eukaryotic tree of life and are poorly represented among reference genomes (Read et al. 2013). The depth of the CCMP1528 transcriptome further explains the annotation rate because lower expressed genes are less likely to be annotated and deeply sequenced transcriptomes capture more genes with low expression (Kuo et al. 2013). This relationship is exemplified by the functional annotation rate for the significantly DE genes being more than double the rate for the whole transcriptome, regardless of annotation method (Table 1).

**Table 1:** Pfam, Gene Ontology (GO), and Kyoto Encyclopedia of Genes and Genomes (KEGG) annotation statistics for the *Phaeocystis globosa* CCMP1528 transcriptome assembly and differentially expressed (DE) genes

	Pfam/GO (%)	KEGG (%)
Total genes annotated	17,826 (26)	9,967 (14)
Total w. pathway or GO	8,962 (13)	5,585 (8)
DE genes annotated	5,180 (66)	3,305 (43)
DE genes w. pathway or GO	2,764 (35)	1,883 (24)

### Differential gene expression analysis

Gene expression patterns in colonial and solitary replicates were explored with a principal component analysis (PCA) and an expression heatmap. Initial data exploration revealed the colonial replicate C2 as an outlier from the rest of the colonial replicates (Fig. S4A, B). The replicate C2 clustered separately from the other three colonial replicates and the four solitary replicates in PCA (Fig. S4A). The heatmap shows C2 gene expression includes similarities with both solitary and colonial replicates: the large number of downregulated genes in the other colonial cultures are not downregulated in C2, but upregulated genes in the other colonial replicates are upregulated in C2 as well (Fig. S4B). These results could be due to high solitary cell density in C2 compared to the other colonial replicates (Fig. 2). The colonial cultures were filtered with 10‐μm pore‐size to limit the inclusion of solitary cells, but higher cell density would inevitably lead to less efficient separation of cell types. Additionally, C2 expressed 26 genes at a higher rate than any of the other replicates. A third of these genes were functionally annotated and involved in abiotic stress, defense, and cell growth, which could be due to the higher cell density observed. For example, genes for peroxidase were among the uniquely upregulated genes in C2 and are important for preventing cell damage from H_2_O_2_ produced in dense algal cultures (Tichy and Vermaas 1999). As a result of these observations, the colonial replicate C2 was excluded from further analyses.

**Figure 3:**
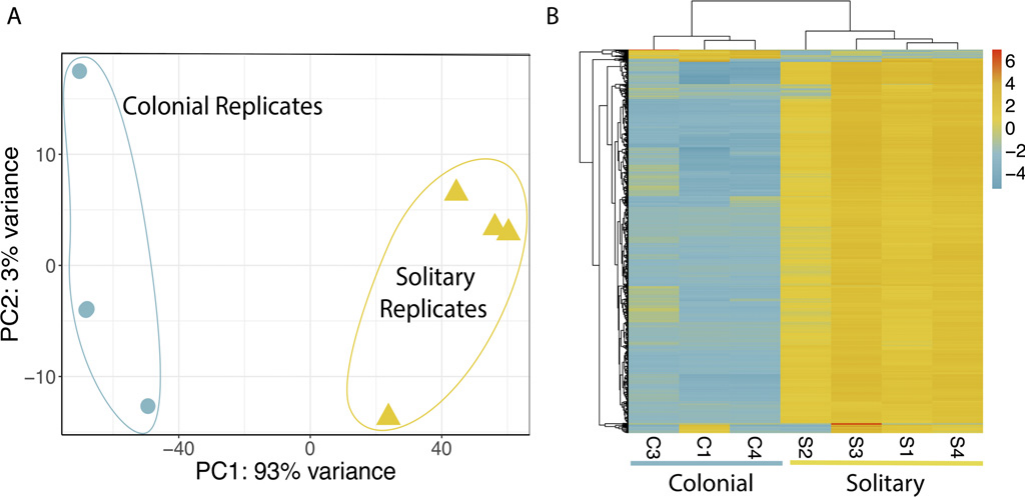
Principal component analysis (PCA) and heatmap demonstrating gene expression patterns in colonial and solitary *Phaeocystis globosa*. **A**. PCA performed on distances between samples derived from regularized log transformed counts. Colonial and solitary replicates cluster separately, and the majority of variance is between sample type rather than within replicates. Results plotted with R package ggplot2. **B**. Heatmap includes the 1,000 significantly differentially expressed genes with the lowest False Discovery Rate (FDR) adjusted *P*‐values. Heatmap color represents difference from the mean regularized log transformed count for each contig in each sample. The majority of differentially expressed genes are downregulated in colonial replicates, and replicates cluster by sample type. Results plotted with R package pheatmap.

The remaining three colonial replicates clustered separately from the four solitary replicates in a PCA plot (Fig. 3A). Differential expression analysis identified 535 genes (0.8%) as significantly upregulated and 7,234 genes (10.4%) as significantly downregulated in colonial replicates. An expression heatmap of the most DE genes sorted by FDR‐adjusted *P*‐value (padj) clearly illustrates the overall expression pattern—the majority of significantly DE genes are downregulated in colonial replicates (Fig. 3B).

### Gene set enrichment analysis

**Figure 4:**
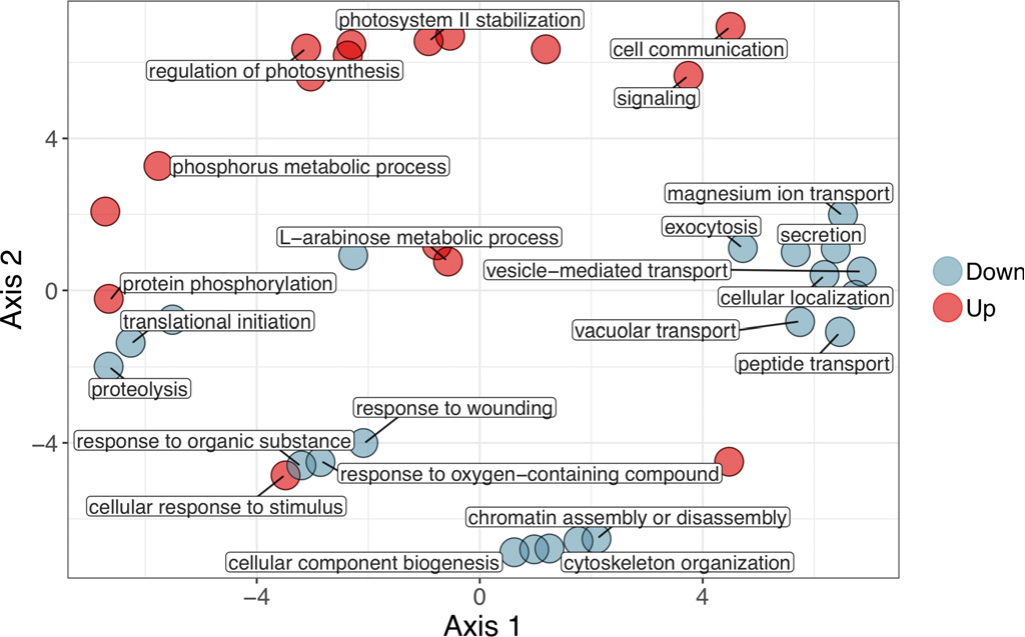
Multidimensional scaling plot of semantic similarities between nonredundant Gene Ontology (GO) terms overrepresented in significantly up‐ and downregulated gene sets. Analysis performed using the REVIGO tool (http://revigo.irb.hr/) with allowed similarity set to 0.7 (to remove redundant GO terms) and the SimRel metric selected to calculate similarities. REVIGO results were exported to the R statistical environment and plotted with ggplot2. Representative GO terms were manually selected and labeled on the plot. To view all GO term labels, an interactive version of the plot made with R package ggplotly is available at: https://brisbin.shinyapps.io/shinycolsol/.

In order to identify Biological Process (BP) GO terms overrepresented in significantly up‐ and downregulated gene sets, we applied a hypergeometric test with a significance cut‐off of *P* < 0.05. Twenty BP GO term were overrepresented among significantly upregulated genes in colonial samples and were primarily involved in cell signal transduction in response to external stimuli (Fig. 4; Table S3). Notably, GO terms involving arabinose, a component of the colonial matrix, were enriched among upregulated genes. In the downregulated gene set, 48 BP GO terms were enriched, including several involved in cation transport, response to oxygen‐containing compounds, translation and protein transport, and vacuolar transport and exocytosis (Fig. 4; Table S4). REVIGO software was used to remove redundant GO terms from lists of enriched terms and to visualize results in a Multidimensional Scaling (MDS) plot (Fig. 4) based on GO term semantic similarities (Supek et al. 2011) as determined by shared ancestry (Pesquita et al. 2009).

When we applied hypergeometric testing to KEGG pathways, only the cGMP‐PKG signaling pathway (cyclic guanosine monophosphate‐protein kinase G pathway) was enriched in the upregulated gene set (*P* < 0.05) (Table S5). Five pathways were enriched among downregulated genes: Lysosome, Autophagy, MAPK signaling pathway (mitogen‐activated protein kinase signaling pathway), AMPK signaling pathway (adenosine monophosphate‐activated protein kinase signaling pathway), and Epidermal growth factor receptor (EGFR) tyrosine kinase inhibitor resistance (Table S6). The lower annotation rate for KEGG pathways compared with GO terms contributed to the difference in enrichment testing results. We therefore applied a linear model test for KEGG pathway enrichment, which identified several additional pathways as being significantly enriched in the up‐ (6) and downregulated (7) gene sets. With this additional test, the PI3K‐Akt signaling (phosphoinositide 3‐kinase‐protein kinase B signaling pathway), Glycosphingolipid biosynthesis, Ferroptosis, Plant–pathogen interaction, Circadian rhythm, and Viral carcinogenesis pathways were also enriched among upregulated genes (Table S7). The Protein processing in endoplasmic reticulum, Oxidative phosphorylation, Ras signaling pathway, Sphingolipid metabolism, Steroid biosynthesis, Fatty acid degradation, and Taste transduction pathways were additionally identified as enriched in downregulated genes (Table S8).

### Genes associated with dimethylsulfoniopropionate and dimethylsulfide production

**Figure 5:**
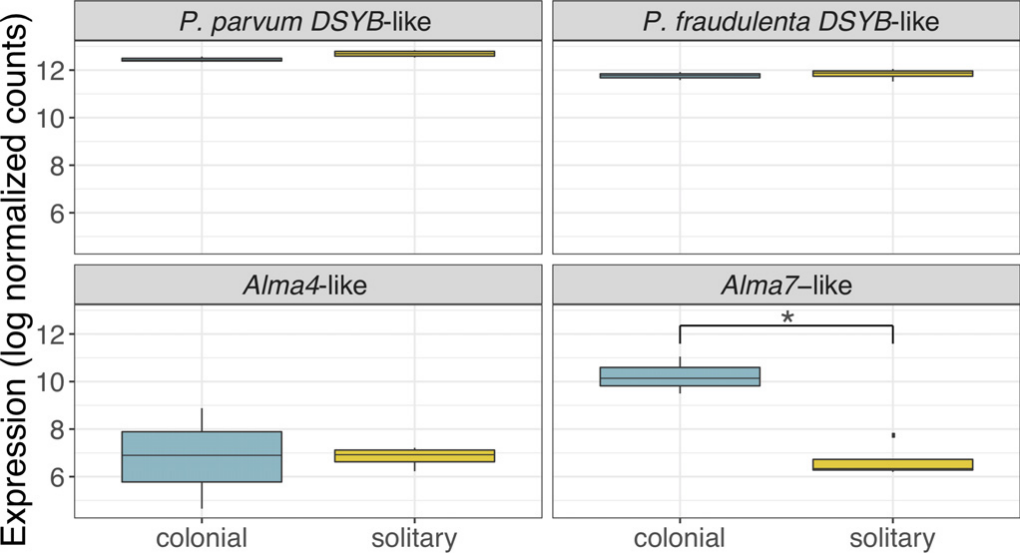
Normalized expression levels of *DSYB*‐like and *Alma*‐like genes in *Phaeocystis globosa* colonial and solitary cell cultures. Box plots show the range, quartiles and median of the log normalized counts for each gene in colonial and solitary culture replicates. Only the *Emiliania huxleyi Alma7*‐like gene was significantly differentially expressed and was upregulated in colonial samples (padj = 1.44E‐12, log fold‐change = 3.55) and significance is indicated with an asterisk (*) on the plot. Results plotted with R package ggplot2.

A blastp query against curated DSYB protein sequences from Curson et al. (2018) and Alma family protein sequences from Alcolombri et al. (2015) identified four *Phaeocystis globosa* contigs as putative *DSYB* or *Alma* family genes (Table 2). The *P. globosa* amino acid (AA) sequence translated from Transcript_30752 aligned with the sequence for *Prymnesium parvum* CCAP946/1B DSYB protein, which is experimentally proven to be highly active. It is therefore likely that this gene is active in DMSP biosynthesis in *Phaeocystis globosa*. A second *P. globosa* AA sequence, from Transcript_31221, aligned with the *Pseudonitzchia fraudulenta* DYSB protein sequence, making it a possible *DSYB* gene as well. The *Pseudonitzchia* DSYB protein has not been experimentally proven to be active, but its sequence is phylogenetically close to the *Fragillariopsis* DSYB, which has proven activity. Neither putative *P. globosa DSYB* genes were DE between solitary and colonial culture replicates but were both expressed at relatively high levels in both sample types (Fig. 5).

**Table 2:** *Phaeocystis globosa* CCMP1528 blastp results against DSYB and Alma family reference sequences (*e*‐values < 1E‐30)

	Database sequence	%ID	Alignment length (AAs)	Gaps	*E‐*value	Bit score
Transcript_30752	*Prymnesium parvum* CCAP946/1B DSYB	74	314	2	1.15E‐170	472
Transcript_31221	*Pseudonitzschia fraudulenta* WWA7 DSYB	45	263	8	1.92E‐65	210
Transcript_36000	*Emiliania huxleyi* Alma7	61	272	4	3.70E‐119	336
Transcript_68879	*Emiliania huxleyi* Alma4	40	253	4	2.64E‐48	166

Two *Alma* family‐like genes were identified in the *Phaeocystis globosa* transcriptome. One *P. globosa* AA sequence, from Transcript_36000, aligned with *Emiliania huxleyi* Alma7. All four *Alma* homologs in the MMETSP *Phaeocystis antarctica* transcriptome are phylogenetically closest to the *E. huxleyi Alma7*, but *E. huxleyi* Alma7 has not been experimentally proven to have DMSP‐lyase activity. A second *P. globosa* AA sequence, from Transcript_68879, aligned with *E. huxleyi* Alma4, which has not been experimentally proven active. Both putative *P. globosa Alma* family genes were expressed at lower rates than *DSYB*‐like genes. The *P. globosa Alma4*‐like gene was not DE in solitary and colonial culture replicates (Fig. 5). The *P. globosa Alma7*‐like gene, however, was significantly upregulated in colonial replicates (padj = 1.44E‐12, logFC = 3.55), suggesting that DMSP biosynthesis is occurring in both colonial and solitary cells, but colonial cells may be cleaving DMSP to DMS and acrylate more actively than solitary cells.

## Discussion

Colonial *Phaeocystis* blooms widely impact ecosystem function and can be extremely detrimental in some systems, particularly in subtropical and tropical coastal regions. Although many hypotheses exist, factors initiating colonial blooms and the ecological function of colonial formation remain enigmatic. We investigated gene expression associated with colony formation in a warm‐water colony‐forming strain of *Phaeocystis globosa* to identify cellular processes associated with colony formation and the functional role of colonies in the *Phaeocystis* life‐cycle. Overall, we observed a transcriptional shift in colonial cultures compared to solitary cell cultures, with vastly more genes significantly downregulated in colonial cells than upregulated (Fig. 3B). This shift suggests that there are trade‐offs associated with colony production and resources must be diverted to construct and maintain the colonial matrix. A relatively small number of genes are upregulated to maintain colonies, but the low annotation rate of these genes, and the transcriptome overall, make it challenging to fully interpret the results (Table 1). Gene set enrichment analyses inherently rely on how many and which genes are annotated and systematic bias in gene annotation influences results (Haynes et al. 2018). However, these analyses still assist in identifying pathways and functions that may be important to the question at hand and indicate genes and pathways that should be followed up in future studies. The results presented here highlight genes involved in constructing the colonial matrix, changes in cellular morphology, responding to external stimuli, cellular proliferation, and producing DMSP, DMS, and acrylate. Results from this study support a resource‐intensive, defensive role for colony formation in *Phaeocystis globosa*.

### Colony matrix carbohydrates and colonial cell morphology

Differential expression of genes associated with clearly observable changes between treatment groups can serve to “ground‐truth” results from RNA‐seq experiments and therefore increase confidence in expression changes detected for genes for less observable traits. In this study, changes in cellular morphology and colony formation itself are clearly observable differences for which several associated genes are DE. The observed expression patterns for these genes can additionally provide new insight into the construction of the colonial matrix and pathways associated with morphological changes in colonial *Phaeocystis* cells. Many different polysaccharides are recognized as contributors to the matrix structure of *Phaeocystis globosa* colonies, including arabinose, rhamnose, xylose, mannose, galactose, glucose, gluconuronate, and O‐methylated pentose sugars (Janse et al. 1996). *Phaeocystis* isolated from different locations tends to have distinct matrix carbohydrate fingerprints, which may be due to genetic attributes of different strains or could arise from different environmental conditions, such as light or nutrient availability. For example, arabinose is the most abundant matrix carbohydrate in *P. globosa* sampled from the North Sea (Janse et al. 1996). In our results, the GO term for arabinose metabolic process was enriched among upregulated genes in colonial cells (Fig. 4; Table S3). These results suggest arabinose is among the dominant matrix polysaccharides in *P. globosa* CCMP1528. The colonial matrix also contains nitrogen (Hamm 2000), which is likely included in amino sugars (Solomon et al. 2003), but we did not find amino sugar biosynthesis or metabolism upregulated in colonial cells.

Divalent cations, particularly Mg^2+^ or Ca^2+^, are required for colonial polymers to gel and contribute to the stability of the colonial matrix (Van Boekel 1992). GO terms for divalent inorganic cation transport, magnesium ion transport, and divalent metal ion transport, however were enriched in downregulated genes in colonial replicates (Fig. 3; Table S4). Similarly, Bender et al. (2018) found that *Phaeocystis antarctica* produces more calcium‐binding proteins when iron limitation suppresses colony formation. These results may be due to the importance of divalent cations for flagellate motility. Actively swimming *Phaeocystis globosa* flagellates, as observed in this study, may require continuous transport of divalent cations to the point of masking their importance in colony formation. Calcium signaling induces secretion of vesicles containing gel‐forming polymers (Chin et al. 2004), which further confounds the observed downregulation of divalent cation transport genes in colonies. However, flagellates also secrete vesicles, but instead of gel polymers they contain star‐shaped structures composed of chitinous filaments (Chrétiennot‐Dinet et al. 1997). The exact function of these structures is unknown, but they may be involved in mating or defense (Dutz and Koski 2006). In addition to divalent cation transport, a number of other GO‐terms enriched in downregulated genes may be involved in secreting these structures, such as exocytosis, secretion, vesicle mediated transport, and vacuolar transport (Fig. 3; Table S3). Alternatively, these GO terms may be involved in scale formation and secretion (Taylor et al. 2007), as scales are only observed on *Phaeocystis globosa* flagellates and not colonial cells (Rousseau et al. 2007).

### A defensive role for colony formation: resource allocation, pathogen interaction, and dimethylsulfide/acrylate production

Out of 7,769 genes that were significantly DE between colonial and solitary replicates, 7,234 genes were downregulated in colonial cells. This dramatic transcriptional shift in colonial cells supports a high resource cost associated with producing colonies (Wang et al. 2015). Specifically, our results indicate that resources are being diverted from protein translation and transport and cell division in order to produce or maintain the colonial matrix. Several GO terms involved in the synthesis of larger nitrogenous compounds and their transport, including translation initiation, protein metabolic process, protein N‐linked glycosylation, and protein transport were significantly enriched in downregulated genes in colonial cells (Fig. 4; Table S4). Similarly, the KEGG pathway, Protein processing in endoplasmic reticulum, was enriched in downregulated genes in colonies (Table S7). Likewise, several mitosis‐associated GO terms (chromatin assembly and disassembly, cytoskeleton organization, cellular component biogenesis) were enriched among downregulated genes in colonial cells (Fig. 3; Table S4). However, the downregulation of mitosis‐associated genes in colonial cells conflicts with observations in previous studies. Veldhuis et al. (2005) observed that colonial cells divide at a higher rate than solitary cells and proposed that in addition to experiencing less grazing and viral lysis, colonial cells may dominate blooms because they outgrow solitary cells. We believe the difference in our results may be due to the type of solitary cells observed. The solitary cells in previous studies could have been diploid flagellates, especially if they were derived from disrupted colonies. The small cell diameter and rapid swimming in solitary cells from our study suggest they are haploid flagellates, which are reported to divide extremely rapidly (Rousseau et al. 2007).

There were several signaling pathways represented in the results that suggest colonial cells are exposed to fewer general stressors and potentially different pathogens than solitary cells. In plants, the MAPK (mitogen‐activated protein kinase) pathway primarily transduces signals from extracellular stressors to the nucleus or cytoplasm and initiates an appropriate response (Taj et al. 2010). In our results, the MAPK signaling pathway was significantly enriched among genes downregulated in colonies. The downregulated genes in this pathway encode MAP3Ks, MAP2Ks, and MAPKs, which are activated in response to pathogen attack and infection, phytohormones, cold and salt stress, and reactive oxygen species (Taj et al. 2010). A decreased stress response in colonies is additionally evidenced by related GO terms enriched among downregulated genes, specifically response to wounding and response to oxygen‐containing compounds. These results support the hypothesis that colony formation serves a defensive purpose. Defense responses such as those regulated by the MAPK pathway are unneeded if the colony skin is mitigating effects from pathogens and abiotic stressors.

Interestingly, the plant–pathogen interaction pathway was enriched among upregulated genes in colonial cells, demonstrating that defensive needs vary between different morphotypes and suggesting that specific pathogen interactions may influence colony formation. Genes upregulated in this pathway encode for calcium‐dependent protein kinases and calcium‐binding protein CML (calmodulin‐like protein), which are immune response components activated following recognition of specific pathogen‐associated molecular patterns (Cheval et al. 2013). Specific bacterial interactions are known to influence transitions between life‐cycle stages in several other protists. For example, specific bacteria stimulate growth in marine diatoms (Amin et al. 2015) and specific bacterial signaling molecules are responsible for inducing both colony formation (Woznica et al. 2016) and sexual reproduction (Woznica et al. 2017) in a species of choanoflagellates, another group of colony‐forming marine protists. In *Phaeocystis*, axenic cultures exhibit decreased growth rates (Solomon et al. 2003), but the effects of specific bacteria on colony formation have not been investigated. In this study, the bacterial community compositions represented in the RNA from the solitary and colonial replicates were significantly different (Fig. S5), but this difference could be partially or completely driven by filtering the sample types through different pore‐sized filters. However, *Spongiibacter—*a genus isolated from marine sponges and found in co‐culture with cyanobacteria*—*is more abundant within colonial samples and is notable for its ability to grow with arabinose, a *Phaeocystis* colonial matrix carbohydrate, as its sole carbon source (Hwang and Cho 2009). While we cannot resolve the effect of bacteria on colony formation, bacteria–*Phaeocystis* interactions represent an interesting avenue for future research.

*Phaeocystis* is a copious producer of DMSP and its cleavage products, DMS and acrylate. DMS and acrylate have been indicated as grazer‐deterrents and antimicrobials (Hamm 2000; Noordkamp et al. 2000; Wolfe and Steinke 1996). While algal genes associated with DMSP biosynthesis (*DSYB*, Curson et al. 2018) and cleavage (*Alma* family genes, Alcolombri et al. 2015) have been identified in many algal transcriptomes, including *Phaeocystis antarctica*, this study is the first to identify these genes for *Phaeocystis globosa*. We found that colonial and solitary *P. globosa* expressed *DSYB*‐like genes at similar levels, suggesting that the two cell types produce similar amounts of DMSP. *DSYB* expression in *Prymnesium parvum*, the haptophyte in which DSYB was discovered, is affected only by salinity, potentially indicating that DMSP production functions primarily in osmoregulation rather than in grazer or pathogen defense (Curson et al. 2018). Similar *DSYB*‐like gene expression levels in colonial and solitary *P. globosa* cells support a basic, shared function for DMSP in the two cell types. Contrastingly, an *Alma* family gene was upregulated in colonial cells. Acrylate accumulates in *Phaeocystis* colonies and may serve to deter grazers and pathogens from disrupting the colonial matrix (Noordkamp et al. 2000). While acrylate may accumulate in colonies simply because it cannot escape through the colonial skin, the upregulation of an *Alma*‐like gene in colonies suggests that colonial *Phaeocystis* cells may actively produce more acrylate and DMS than solitary cells, providing additional support for colony formation serving a defensive role in *P. globosa*.

### Role of colonies in *Phaeocystis* reproduction

Colony formation is hypothesized to be involved in sexual reproduction in *Phaeocystis* since swarming flagellates have been observed within senescent colonies (Peperzak et al. 2000; Rousseau et al. 2013). However, we did not find meiosis or sexual reproduction GO terms or KEGG pathways enriched in up‐ (or down‐) regulated genes. These results may arise from RNA being extracted during late exponential growth phase. Previous observations suggest that colonies produce flagellates during bloom decay, so we might have found meiosis genes upregulated in colonial cells if we had sampled during the late stationary phase instead.

### Signaling pathways associated with colony formation

Processes and pathways involved in cell‐signaling, cell communication, and response to stimuli that are enriched in upregulated‐genes are particularly interesting because they shed some light on factors promoting colony formation and maintenance in *Phaeocystis globosa*. The cGMP‐PKG signaling pathway was the only KEGG pathway significantly enriched among upregulated genes when a hypergeometric test was used. Three genes upregulated in this pathway encoded: (1) cGMP‐dependent protein kinase, which phosphorylates biologically important targets, has been implicated in cell division and nucleic acid synthesis, and reduces cytoplasmic Ca^2+^ concentrations (Lincoln et al. 2001); (2) cAMP‐dependent protein kinase regulator; and (3) a cAMP‐responsive element‐binding protein (CREB), which binds to DNA to increase or decrease transcription and is associated with increased cell survival (Chrivia et al. 1993). The PI3K‐Akt signaling pathway was also significantly enriched in upregulated genes when the additional linear model test was used. Within this pathway, two extracellular matrixfocal adhesion genes, for tenascin (a glycoprotein) and type IV collagen, were significantly upregulated. Focal adhesion proteins connect cells to extracellular matrices both literally and figuratively, by holding cells in place and by initiating cellular responses to external conditions (Wozniak et al. 2004). Likewise, Bender et al. (2018) found genes for focal adhesion proteins, specifically glycoproteins, upregulated in colonial *Phaeocystis antarctica*. It is therefore likely that these proteins have an important function in structurally maintaining cell positions in the colonial matrix and signaling between colonial cells. Focal adhesion proteins may be mediating interactions with protein kinases in colonial cells, which go on to promote cell proliferation and differentiation into the colonial morphotype. These signaling pathways represent important candidates for continued study of molecular mechanisms regulating colony formation.

### Conclusions and future directions

This study investigated gene expression associated with colony formation in *Phaeocystis globosa* for the first time and discovered a large transcriptional shift associated with colony production. Differentially expressed genes were mostly downregulated in colonies, providing evidence for extensive resource allocation toward colony formation. Together, changes in the types of active pathogen interaction pathways, reduced expression of stress‐response pathways, and increased expression of a DMSP‐lyase, which produces DMS and acrylate, support a defensive role for colony formation. Future studies may extend this work by investigating *P. globosa* gene expression in colonial and solitary cells in a time course study through the waxing and waning of a bloom and under different nutrient and grazing regimes, potentially using mesocosms or metatranscriptomic methods in natural communities. While our ability to fully interpret the results was inhibited by an overall lack of annotated genomes and transcriptomes for diverse protist lineages, this study contributes a new and deeply sequenced transcriptome for *Phaeocystis globosa*. Identification of *DSYB* and *Alma* family‐like genes in this transcriptome will allow for further investigation into *P. globosa* DMSP and DMS production in the oceans. Additionally, we identified several protein kinase signaling pathways that are potentially important for regulating colony formation and should be experimentally investigated in follow‐up studies. The results presented here will guide and facilitate continued efforts to unravel the complex factors involved in initiating harmful colonial *Phaeocystis* blooms, which will likely increase with continued climate change and nutrient pollution in the future.

## Supporting information


**Figure S1.** Phylogenetic placement of *Phaeocystis globosa* strains CCMP1528 and CCMP2170.
**Figure S2.** Correlations between External RNA Controls Consortium (ERCC) standard sequences’ initial concentrations and FPKM in colonial and solitary replicates.
**Figure S3.** Percent of eukaryote and protist Benchmarking Universal Single Copy Orthologs (BUSCOs) complete, fragmented, or missing in *Phaeocystis globosa* CCMP1528 and *Phaeocystis* sp. CCMP2710 transcriptomes.
**Figure S4.** Principal component analysis (PCA) and heatmap demonstrating gene expression patterns in colonial and solitary *Phaeocystis globosa*, including outlier sample C2.
**Figure S5.** Relative abundance of bacterial 16S rRNA sequences present in the RNA extracted from colonial and solitary culture replicates.
**Table S1.** Sequencing statistics for each sample before and after quality filtering.
**Table S2.** Summary statistics for MMETSP *Phaeocystis* sp. CCMP2710 transcriptome and *Phaeocystis globosa* CCMP1528 transcriptome assembly.
**Table S3.** Biological Process Gene Ontology (GO) terms overrepresented in significantly upregulated genes in colonial cultures: results from GOStats hypergeometric test.
**Table S4.** Biological Process Gene Ontology (GO) terms overrepresented in significantly downregulated genes in colonial cultures: results from GOStats hypergeometric test.
**Table S5**. KEGG pathways overrepresented in significantly upregulated genes in colonial cultures: results from ClusterProfiler enricher test.
**Table S6.** KEGG pathways overrepresented in significantly downregulated genes in colonial cultures: results from ClusterProfiler enricher test.
**Table S7.** KEGG pathways overrepresented in significantly upregulated genes in colonial cultures: results from edgeR kegga test.
**Table S8.** KEGG pathways overrepresented in significantly downregulated genes in colonial cultures: results from edgeR kegga test.

## References

[jeu12727-bib-0001] Alcolombri, U. , Ben‐dor, S. , Feldmesser, E. , Levin, Y. , Tawfik, D. S. & Vardi, A. 2015 Identification of the algal dimethyl sulfide‐releasing enzyme: a missing link in the marine sulfur cycle. Science, 348:1–4. 10.1126/science.aab1586 26113722

[jeu12727-bib-0002] Amin, S. A. , Hmelo, L. R. , van Tol, H. M. , Durham, B. P. , Carlson, L. T. , Heal, K. R. , Morales, R. L. , Berthiaume, C. T. , Parker, M. S. , Djunaedi, B. , Ingalls, A. E. , Parsekm, M. R. , Moran, M. A. & Ambrust, E. V. 2015 Interaction and signalling between a cosmopolitan phytoplankton and associated bacteria. Nature, 522:98–101. 26017307 10.1038/nature14488

[jeu12727-bib-0003] Andersen, R. A. , Bailey, J. C. , Decelle, J. & Probert, I. 2015 *Phaeocystis rex* sp. nov. (Phaeocystales, Prymnesiophyceae): a new solitary species that produces a multilayered scale cell covering. Eur. J. Phycol., 50:207–222.

[jeu12727-bib-0004] Andrews, S. 2010 FastQC: a quality control tool for high throughput sequence data. Available online at: http://www.bioinformatics.babraham.ac.uk/projects/fastqc.

[jeu12727-bib-0005] Bender, S. J. , Moran, D. M. , McIlvin, M. R. , Zheng, H. , McCrow, J. P. , Badger, J. , DiTullio, G. R. , Allen, A. E. & Saito, M. A. 2018 Iron triggers colony formation in *Phaeocystis antarctica*: connecting molecular mechanisms with iron biogeochemistry. Biogeosciences, 15:4923–4942.

[jeu12727-bib-0006] Bolger, A. M. , Lohse, M. & Usadel, B. 2014 Trimmomatic: a flexible trimmer for Illumina sequence data. Bioinformatics, 30:2114–2120. 24695404 10.1093/bioinformatics/btu170 PMC4103590

[jeu12727-bib-0007] Brussaard, C. P. , Bratbak, G. , Baudoux, A. C. & Ruardij, P. 2007 *Phaeocystis* and its interaction with viruses In: Phaeocystis, major link in the biogeochemical cycling of climate‐relevant elements. Springer, Dordrecht p. 201‐215.

[jeu12727-bib-0008] Brussaard, C. P. D. , Kuipers, B. & Veldhuis, M. J. W. 2005 A mesocosm study of *Phaeocystis globosa* population dynamics: I. Regulatory role of viruses in bloom control. Harmful Algae, 4:859–874.

[jeu12727-bib-0009] Camacho, C. , Coulouris, G. , Avagyan, V. , Ma, N. , Papadopoulos, J. , Bealer, K. & Madden, T. J. 2009 BLAST+: architecture and applications. BMC Bioinformatics, 10:1–9. 20003500 10.1186/1471-2105-10-421 PMC2803857

[jeu12727-bib-0010] Cariou, V. , Casotti, R. , Birrien, J. L. & Vaulot, D. 1994 The initiation of *Phaeocystis* colonies. J. Plankton Res., 16:457–470.

[jeu12727-bib-0011] Caron, D. A. , Alexander, H. , Allen, A. E. , Archibald, J. M. , Armbrust, E. V. , Bachy, C. , Bell, C. J. , Bharti, A. , Dyhrman, A. T. , Guida, S. M. , Heidelberg, K. B. , Kaye, J. Z. , Metzner, J. , Smith, S. R. & Worden, A. Z. 2017 Probing the evolution, ecology and physiology of marine protists using transcriptomics. Nat. Rev. Microbiol., 15:6–20. 27867198 10.1038/nrmicro.2016.160

[jeu12727-bib-0012] Charlson, R. J. , Lovelock, J. E. , Andreae, M. O. & Warren, S. G. 1987 Oceanic phytoplankton, atmospheric sulphur, cloud albedo and climate. Nature, 326:655–661.

[jeu12727-bib-0013] Chen, Y. Q. , Wang, N. , Zhang, P. , Zhou, H. & Qu, L. H. 2002 Molecular evidence identifies bloom‐forming *Phaeocystis* (Prymnesiophyta) from coastal waters of southeast China as *Phaeocystis globosa* . Biochem. Syst. Ecol., 30:15–22.

[jeu12727-bib-0014] Cheval, C. , Aldon, D. , Galaud, J.‐P. & Ranty, B. 2013 Calcium/calmodulin‐mediated regulation of plant immunity. Biochim. Biophys. Acta Mol. Cell Res., 1833:1766–1771. 10.1016/j.bbamcr.2013.01.031 23380707

[jeu12727-bib-0015] Chin, W. C. , Orellana, M. V. , Quesada, I. & Verdugo, P. 2004 Secretion in unicellular marine phytoplankton: demonstration of regulated exocytosis in P*haeocystis globosa* . Plant Cell Physiol., 45:535–542. 15169935 10.1093/pcp/pch062

[jeu12727-bib-0016] Chrétiennot‐Dinet, M.‐J. , Giraud‐Guille, M.‐M. , Vaulot, D. , Putaux, J.‐L. , Saito, Y. & Chanzy, H. 1997 The chitinous nature of filaments ejected by *Phaeocystis* (Prymnesiophyceae). J. Phycol., 33:666–672.

[jeu12727-bib-0017] Chrivia, J. C. , Kwok, R. P. S. , Lamb, N. , Hagiwara, M. , Montminy, M. R. & Goodman, R. H. 1993 Phosphorylated CREB binds specifically to the nuclear protein CBP. Nature, 365:855–859. 8413673 10.1038/365855a0

[jeu12727-bib-0018] Cronin, M. , Ghosh, K. , Sistare, F. , Quackenbush, J. , Vilker, V. & O'Connell, C. 2004 Universal RNA reference materials for gene expression. Clin. Chem., 50:1464–1471. 15155546 10.1373/clinchem.2004.035675

[jeu12727-bib-0019] Curson, A. R. J. , Williams, B. T. , Pinchbeck, B. J. , Sims, L. P. , Martínez, A. B. , Rivera, P. P. L. , Kumaresan, D. , Mercade, E. , Spurgin, L. G. , Carrion, O. , Moxon, S. , Cattolico, R. A. , Kuzhiumparambil, U. , Guagliardo, P. , Clode, P. L. , Raina, J.‐B. & Todd, J. D. 2018 DSYB catalyses the key step of dimethylsulfoniopropionate biosynthesis in many phytoplankton. Nat. Microbiol, 3:430–439. 29483657 10.1038/s41564-018-0119-5

[jeu12727-bib-0020] Desroy, N. & Denis, L. 2004 Influence of spring phytodetritus sedimentation on intertidal macrozoobenthos in the eastern English Channel. Mar. Ecol. Prog. Ser., 270:41–53.

[jeu12727-bib-0021] Doan‐Nhu, H. , Nguyen‐Ngoc, L. & Dippner, J. W. 2010 Development of *Phaeocystis globosa* blooms in the upwelling waters of the South Central coast of Vietnam. J. Mar. Syst., 83:253–261.

[jeu12727-bib-0022] Dutz, J. & Koski, M. 2006 Trophic significance of solitary cells of the prymnesiophyte *Phaeocystis globosa* depends on cell type. Limnol. Oceanogr., 51:1230–1238.

[jeu12727-bib-0023] Eddy, S. R. 2011 Accelerated profile HMM searches. PLoS Comput. Biol., 7:e1002195. 22039361 10.1371/journal.pcbi.1002195 PMC3197634

[jeu12727-bib-0024] Falcon, S. & Gentleman, R. 2007 Using GOstats to test gene lists for GO term association. Bioinformatics, 23:257–258. 17098774 10.1093/bioinformatics/btl567

[jeu12727-bib-0500] Finn, R. D. , Coggill, P. , Eberhardt, R. Y. , Eddy, S. R. , Mistry, J. , Mitchell, A. L. , Potter, S. C. , Punta, M. , Qureshi, M. , Sangrador‐Vegas, A. & Salazar, G. A. 2015 The Pfam protein families database: towards a more sustainable future. Nucleic acids research, 44:D279‐D285. 26673716 10.1093/nar/gkv1344 PMC4702930

[jeu12727-bib-0025] Fu, L. , Niu, B. , Zhu, Z. , Wu, S. & Li, W. 2012 CD‐HIT: accelerated for clustering the next‐generation sequencing data. Bioinformatics, 28:3150–3152. 23060610 10.1093/bioinformatics/bts565 PMC3516142

[jeu12727-bib-0026] Gaebler‐Schwarz, S. , Davidson, A. , Assmy, P. , Chen, J. , Henjes, J. , Nöthig, E. M. , Lunau, M. & Medlin, L. K. 2010 A new cell stage in the haploid‐diploid life cycle of the colony‐forming haptophyte *Phaeocystis antarctica* and its ecological implications. J. Phycol., 46:1006–1016.

[jeu12727-bib-0027] Grabherr, M. G. , Haas, B. J. , Yassour, M. , Levin, J. Z. , Thompson, D. A. , Amit, I. , Adiconis, X. , Fan, L. , Raychowdhury, R. , Zeng, Q. , Chen, Z. , Mauceli, E. , Hacohen, N. , Gnirke, A. , Rhind, N. , di Palma, F. , Birren, B. W. , Nusbaum, C. , Lindblad‐Toh, K. , Friedman, N. & Regev, A. 2013 Trinity: reconstructing a full‐length transcriptome without a genome from RNA‐Seq data. Nat. Biotechnol., 29:644–652. 10.1038/nbt.1883 PMC3571712 21572440

[jeu12727-bib-0028] Haas, B. J. , Papanicolaou, A. , Yassour, M. , Grabherr, M. , Philip, D. , Bowden, J. , Couger, M. B. , Eccles, D. , Li, B. , Lieber, M. , MacManes, M. D. , Ott, M. , Orvis, J. , Pochet, N. , Strozi, F. , Weeks, N. , Westerman, R. , William, T. , Dewey, C. N. , Henschel, R. , LeDuc, R. D. , Friedman, N. & Regev, A. 2013 De novo transcript sequence reconstruction from RNA‐seq: reference generation and analysis with Trinity. Nat. Protoc., 8:1–43. 23845962 10.1038/nprot.2013.084 PMC3875132

[jeu12727-bib-0029] Hamm, C. E. 2000 Architecture, ecology and biogeochemistry of *Phaeocystis* colonies. J. Sea Res., 43:307–315.

[jeu12727-bib-0030] Haynes, W. A. , Tomczak, A. & Khatri, P. 2018 Gene annotation bias impedes biomedical research. Sci. Rep., 8:1–7. 29358745 10.1038/s41598-018-19333-x PMC5778030

[jeu12727-bib-0031] Houdan, A. , Billard, C. , Marie, D. , Not, F. , Sâez, A. G. , Young, J. R. & Probert, I. 2004 Holococcolithophore‐ heterococcolithophore (Haptophyta) life cycles: flow cytometric analysis of relative ploidy levels. Syst. Biodivers., 1:453–465.

[jeu12727-bib-0032] Hwang, C. Y. & Cho, B. C. 2009 *Spongiibacter tropicus* sp. nov., isolated from a *Synechococcus* culture. Int. J. Syst. Evol. Microbiol., 59:2176–2179. 19605732 10.1099/ijs.0.005819-0

[jeu12727-bib-0033] Janse, I. , van Rijssel, M. , Gottschal, Jan. C. , Lancelot, C. & Gieskes, W. W. C. 1996 Carbohydrates in the North Sea during spring bloom of *Phaeocystis*: a specific fingerprint. Aquat. Microb. Ecol., 10:97–103.

[jeu12727-bib-0034] Kanehisa, M. , Sato, Y. & Morishima, K. 2016 BlastKOALA and GhostKOALA: KEGG tools for functional characterization of genome and metagenome sequences. J. Mol. Biol., 428:726–731. 26585406 10.1016/j.jmb.2015.11.006

[jeu12727-bib-0035] Keeling, P. J. , Burki, F. , Wilcox, H. M. , Allam, B. , Allen, E. E. , Amaral‐Zettler, L. A. , Armbrust, E. V. , Archibald, J. M. , Bharti, A. K. , Bell, C. J. , Beszteri, B. , Bidle, K. D. , Cameron, C. T. , Campbell, L. , Caron, D. A. , Cattolico, R. A. , Collier, J. L. , Coyne, K. , Davy, S. K. , Deschamps, P. , Dyhrman, S. T. , Edvardsen, B. , Gates, R. D. , Gobler, C. J. , Greenwood, S. J. , Guida, S. M. , Jacobi, J. L. , Jakobsen, K. S. , James, E. R. , Jenkins, B. , John, U. , Johnson, M. D. , Juhl, A. R. , Kamp, A. , Katz, L. A. , Kiene, R. , Kudryavtsev, A. , Leander, B. S. , Lin, S. , Lovejoy, C. , Lynn, D. , Marchetti, A. , McManus, G. , Nedelcu, A. M. , Menden‐Deuer, S. , Miceli, C. , Mock, T. , Montresor, M. , Moran, M. A. , Murray, S. , Nadathur, G. , Nagai, S. , Ngam, P. B. , Palenik, B. , Pawlowski, J. , Petroni, G. , Piganeau, G. , Posewitz, M. C. , Rengefors, K. , Romano, G. , Rumpho, M. E. , Rynearson, T. , Schilling, K. B. , Schroeder, D. C. , Simpson, A. G. , Slamovits, C. H. , Smith, D. R. , Smith, G. J. , Smith, S. R. , Sosik, H. M. , Stief, P. , Theriot, E. , Twary, S. N. , Umale, P. E. , Vaulot, D. , Wawrik, B. , Wheeler, G. L. , Wilson, W. H. , Xu, Y. , Zingone, A. & Worden, A. Z. 2014 The marine microbial eukaryote transcriptome sequencing project (MMETSP): illuminating the functional diversity of eukaryotic life in the oceans through transcriptome sequencing. PLoS Biol., 12:e1001889. 24959919 10.1371/journal.pbio.1001889 PMC4068987

[jeu12727-bib-0036] Kuo, R. C. , Zhang, H. , Zhuang, Y. , Hannick, L. & Lin, S. 2013 Transcriptomic study reveals widespread spliced leader trans‐splicing, Short 5 9 ‐UTRs and potential complex carbon fixation mechanisms in the euglenoid alga. PLoS ONE, 8:e60826. 23585853 10.1371/journal.pone.0060826 PMC3621762

[jeu12727-bib-0037] Lauritano, C. , De Luca, D. , Ferrarini, A. , Avanzato, C. , Minio, A. , Esposito, F. & Ianora, A. 2017 *De novo* transcriptome of the cosmopolitan dinoflagellate *Amphidinium carterae* to identify enzymes with biotechnological potential. Sci. Rep., 7:11701. 28916825 10.1038/s41598-017-12092-1 PMC5601461

[jeu12727-bib-0038] Li, B. & Dewey, C. N. 2011 RSEM: accurate transcript quantification from RNA‐Seq data with or without a reference genome. BMC Bioinformatics, 12:323. 21816040 10.1186/1471-2105-12-323 PMC3163565

[jeu12727-bib-0501] Li, H. , Handsaker, B. , Wysoker, A. , Fennell, T. , Ruan, J. , Homer, N. , Marth, G. , Abecasis, G. & Durbin, R. 2009 The sequence alignment/map format and SAMtools. Bioinformatics, 25:2078‐2079. 19505943 10.1093/bioinformatics/btp352 PMC2723002

[jeu12727-bib-0039] Lincoln, T. M. , Dey, N. & Sellak, H. 2001 Signal transduction in smooth muscle invited review: cGMP‐dependent protein kinase signaling mechanisms in smooth muscle: from the regulation of tone to gene expression. J. Appl. Physiol., 91:1421–1430. 11509544 10.1152/jappl.2001.91.3.1421

[jeu12727-bib-0040] Liss, P. S. , Malin, G. , Turner, S. M. & Holligan, P. M. 1994 Dimethyl sulphide and *Phaeocystis*: a review. J. Mar. Syst., 5:41–53.

[jeu12727-bib-0041] Liu, H.‐X. , Huang, H.‐H. , Xu, S.‐N. , Dai, M. & Shen, P.‐P. 2015 Planktonic community structure during a harmful bloom of *Phaeocystis globosa* in a subtropical bay, with special reference to the ciliate assemblages. Ecotoxicology, 24:1419–1429. 25967937 10.1007/s10646-015-1464-2

[jeu12727-bib-0042] Long, J. D. , Smalley, G. W. , Barsby, T. , Anderson, J. T. & Hay, M. E. 2007 Chemical cues induce consumer‐specific defenses in a bloom‐forming marine phytoplankton. Proc. Natl Acad. Sci., 104:10512–10517. 17563379 10.1073/pnas.0611600104 PMC1965544

[jeu12727-bib-0043] Love, M. I. , Huber, W. & Anders, S. 2014 Moderated estimation of fold change and dispersion for RNA‐seq data with DESeq2. Genome Biol., 15:1–21. 10.1186/s13059-014-0550-8 PMC4302049 25516281

[jeu12727-bib-0044] Mitchell, A. , Chang, H.‐Y. , Daugherty, L. , Fraser, M. , Hunter, S. , Lopez, R. , McAnulla, C. , McMenamin, C. , Nuka, G. , Pesseat, S. , Sangrador‐Vegas, A. , Scheremetjew, M. , Rato, C. , Yong, S.‐Y. , Bateman, A. , Punta, M. , Attwood, T. K. , Sigrist, C. J. A. , Redaschi, N. , Rivoire, C. , Xenarios, I. , Kahn, D. , Guyot, D. , Bork, P. , Letunic, I. , Gough, J. , Oates, M. , Haft, D. , Huang, H. , Natale, D. A. , Wu, C. H. , Orengo, C. , Sillitoe, I. , Mi, H. , Thomas, P. D. & Finn, R. D. 2015 The InterPro protein families database: the classification resource after 15 years. Nucleic Acids Res., 43:213–221. 10.1093/nar/gku1243 PMC4383996 25428371

[jeu12727-bib-0045] Noordkamp, D. J. B. , Gieskes, W. W. C. , Gottschal, J. C. , Forney, L. J. & Van Rijssel, M. 2000 Acrylate in *Phaeocystis* colonies does not affect the surrounding bacteria. J. Sea Res., 43:287–296.

[jeu12727-bib-0046] Peng, X. C. , Yang, W. D. , Liu, J. S. , Peng, Z. Y. , Lü, S. H. & Ding, W. Z. 2005 Characterization of the hemolytic properties of an extract from *Phaeocystis globosa* Scherffel. J. Integr. Plant Biol., 47:165–171.

[jeu12727-bib-0047] Peperzak, L. , Colijn, F. , Vrieling, E. G. , Gieskes, W. W. C. & Peeters, J. C. H. 2000 Observations of flagellates in colonies of *Phaeocystis globosa* (Prymnesiophyceae); a hypothesis for their position in the life cycle. J. Plankton Res., 22:2181–2203.

[jeu12727-bib-0048] Peperzak, L. & Gabler‐Schwarz, S. 2012 Current knowledge of the life cycles of *Phaeocystis globosa* and *Phaeocystis antarctica* (prymnesiophyceae). J. Phycol., 48:514–517. 27011066 10.1111/j.1529-8817.2012.01136.x

[jeu12727-bib-0049] Peperzak, L. & Poelman, M. 2008 Mass mussel mortality in The Netherlands after a bloom of *Phaeocystis globosa* (prymnesiophyceae). J. Sea Res., 60:220–222.

[jeu12727-bib-0050] Pesquita, C. , Faria, D. , Falcão, A. O. , Lord, P. & Couto, F. M. 2009 Semantic similarity in biomedical ontologies. PLoS Comput. Biol., 5:e1000443. 19649320 10.1371/journal.pcbi.1000443 PMC2712090

[jeu12727-bib-0051] Quinlan, A. R. & Hall, I. M. 2010 BEDTools: a flexible suite of utilities for comparing genomic features. Bioinformatics, 26:841–842. 20110278 10.1093/bioinformatics/btq033 PMC2832824

[jeu12727-bib-0052] R Core Team . 2013 R: A Language and Environment for Statistical Computing. R Foundation for Statistical Computing, Vienna, Austria http://www.r-project.org/.

[jeu12727-bib-0053] Read, B. A. , Kegel, J. , Klute, M. J. , Kuo, A. , Lefebvre, S. C. , Maumus, F. , Mayer, C. , Miller, J. , Monier, A. , Salamov, A. , Young, J. , Aguilar, M. , Claverie, J. M. , Frickenhaus, S. , Gonzalez, K. , Herman, E. K. , Lin, Y. C. , Napier, J. , Ogata, H. , Sarno, A. F. , Shmutz, J. , Schroeder, D. , de Vargas, C. , Verret, F. , von Dassow, P. , Valentin, K. , Van de Peer, Y. , Wheeler, G. ; Emiliania huxleyi Annotation Consortium , Dacks, J. B. , Delwiche, C. F. , Dyhrman, S. T. , Glöckner, G. , John, U. , Richards, T. , Worden, A. Z. , Zhang, X. & Grigoriev, I. V. 2013 Pan genome of the phytoplankton *Emiliania* underpins its global distribution. Nature, 499(7457):209. 23760476 10.1038/nature12221

[jeu12727-bib-0054] Robinson, M. D. , McCarthy, D. J. & Smyth, G. K. 2009 edgeR: a Bioconductor package for differential expression analysis of digital gene expression data. Bioinformatics, 26:139–140. 19910308 10.1093/bioinformatics/btp616 PMC2796818

[jeu12727-bib-0055] Rousseau, V. , Chrétiennot‐Dinet, M.‐J. , Jacobsen, A. , Verity, P. & Whipple, S. 2007 The life cycle of *Phaeocystis*: state of knowledge and presumptive role in ecology. Biogeochemistry, 83:29–47.

[jeu12727-bib-0056] Rousseau, V. , Lantoine, F. , Rodriguez, F. , LeGall, F. , Chretiennot‐Dinet, M. J. & Lancelot, C. 2013 Characterization of *Phaeocystis globosa* (Prymnesiophyceae), the blooming species in the Southern North Sea. J. Sea Res., 76:105–113.

[jeu12727-bib-0057] Santoferrara, L. F. , Guida, S. , Zhang, H. & McManus, G. B. 2014 *De novo* transcriptomes of a mixotrophic and a heterotrophic ciliate from marine plankton. PLoS ONE, 7:e101418. 10.1371/journal.pone.0101418 PMC4077812 24983246

[jeu12727-bib-0058] Schoemann, V. , Becquevort, S. , Stefels, J. , Rousseau, V. & Lancelot, C. 2005 *Phaeocysti*s blooms in the global ocean and their controlling mechanisms: a review. J. Sea Res., 53:43–66.

[jeu12727-bib-0059] Simão, F. A. , Waterhouse, R. M. , Ioannidis, P. , Kriventseva, E. V. & Zdobnov, E. M. 2015 BUSCO: assessing genome assembly and annotation completeness with single‐copy orthologs. Bioinformatics, 31:3210–3212. 26059717 10.1093/bioinformatics/btv351

[jeu12727-bib-0060] Solomon, C. M. , Lessard, E. J. , Keil, R. G. & Foy, M. S. 2003 Characterization of extracellular polymers of *Phaeocystis globosa* and *P. antarctica* . Mar. Ecol. Prog. Ser., 250:81–89.

[jeu12727-bib-0061] Spilmont, N. , Denis, L. , Artigas, L. F. , Caloin, F. , Courcot, L. , Créach, A. , Desroy, N. , Gevaert, F. , Hacquebart, P. , Hubas, C. , Janquin, M.‐A. , Lemoine, Y. , Luczak, C. , Migne, A. , Rauch, M. & Davoult, D. 2009 Impact of the *Phaeocystis globosa* spring bloom on the intertidal benthic compartment in the eastern English Channel: a synthesis. Mar. Pollut. Bull., 58:55–63. 18947841 10.1016/j.marpolbul.2008.09.007

[jeu12727-bib-0502] Sunda, W. , Kieber, D. J. , Kiene, R. P. & Huntsman, S. 2002 An antioxidant function for DMSP and DMS in marine algae. Nature, 418:317. 12124622 10.1038/nature00851

[jeu12727-bib-0062] Supek, F. , Bošnjak, M. , Škunca, N. & Šmuc, T. 2011 REVIGO summarizes and visualizes long lists of Gene Ontology terms. PLoS ONE, 6:e0021800. 10.1371/journal.pone.0021800 PMC3138752 21789182

[jeu12727-bib-0063] Taj, G. , Agarwal, P. , Grant, M. & Kumar, A. 2010 MAPK machinery in plants. Plant Signal. Behav., 5:1370–1378. 20980831 10.4161/psb.5.11.13020 PMC3115236

[jeu12727-bib-0064] Tang, K. W. 2003 Grazing and colony size development in *Phaeocysis globosa* (Prymnesiophyceae): the role of chemical signal. J. Plankton Res., 35:831–842.

[jeu12727-bib-0065] Tang, K. W. , Jakobsen, H. H. & Visser, A. W. 2001 *Phaeocystis globosa* (Prymnesiophyceae) and the planktonic food web: feeding, growth, and trophic interactions among grazers. Limnol. Oceanogr., 46:1860–1870.

[jeu12727-bib-0066] Taylor, A. R. , Russell, M. A. , Harper, G. M. , Collins, T. F. T. & Brownlee, C. 2007 Dynamics of formation and secretion of heterococcoliths by *Coccolithus pelagicus* ssp. braarudii. Eur. J. Phycol., 42:125–136.

[jeu12727-bib-0067] Tichy, M. & Vermaas, W. 1999 In Vivo role of catalase‐peroxidase in *Synechocystis* sp. strain PCC 6803. J. Bacteriol., 181:1875–1882. 10074082 10.1128/jb.181.6.1875-1882.1999 PMC93588

[jeu12727-bib-0068] Van Boekel, W. H. M. 1992 *Phaeocystis* colony mucus components and the importance of calcium ions for colony stability. Mar. Ecol. Prog. Ser., 87:301–305.

[jeu12727-bib-0069] Van Duyl, F. C. , Gieskes, W. W. C. , Kop, A. J. & Lewis, W. E. 1998 Biological control of short‐term variations in the concentration of DMSP and DMS during a *Phaeocystis* spring bloom. J. Sea Res., 40:221–231.

[jeu12727-bib-0070] Veldhuis, M. J. W. , Brussaard, C. P. D. & Noordeloos, A. A. M. 2005 Living in a *Phaeocystis* colony: a way to be a successful algal species. Harmful Algae, 4:841–858.

[jeu12727-bib-0071] Verity, P. G. & Medlin, L. K. 2003 Observations on colony formation by the cosmopolitan phytoplankton genus *Phaeocystis* . J. Mar. Syst., 43:153–164.

[jeu12727-bib-0072] Wang, X. , Wang, Y. , Ou, L. , He, X. & Chen, D. 2015 Allocation costs associated with induced defense in *Phaeocystis globosa* (Prymnesiophyceae): the effects of nutrient availability. Sci. Rep., 5:10850. 26040243 10.1038/srep10850 PMC4455181

[jeu12727-bib-0073] Wang, X. , Wang, Y. & Smith, W. O. 2011 The role of nitrogen on the growth and colony development of *Phaeocystis globosa* (Prymnesiophyceae). Eur. J. Phycol., 46:305–314.

[jeu12727-bib-0074] Wolfe, G. V. & Steinke, M. 1996 Grazing‐activated production of dimethyl sulfide (DMS) by two clones of *Emiliania huxleyi* . Limnol. Oceanogr., 41:1151–1160.

[jeu12727-bib-0075] Wozniak, M. A. , Modzelewska, K. , Kwong, L. & Keely, P. J. 2004 Focal adhesion regulation of cell behavior. Biochim. Biophys. Acta Mol. Cell Res., 1692:103–119. 10.1016/j.bbamcr.2004.04.007 15246682

[jeu12727-bib-0076] Woznica, A. , Cantley, A. M. , Beemelmanns, C. , Freinkman, E. , Clardy, J. & King, N. 2016 Bacterial lipids activate, synergize, and inhibit a developmental switch in choanoflagellates. Proc. Natl Acad. Sci., 113:7894–7899. 27354530 10.1073/pnas.1605015113 PMC4948368

[jeu12727-bib-0077] Woznica, A. , Gerdt, J. P. , Hulett, R. E. , Clardy, J. & King, N. 2017 Mating in the closest living relatives of animals is induced by a bacterial chondroitinase. Cell, 170:1175–1183.e11. 28867285 10.1016/j.cell.2017.08.005 PMC5599222

[jeu12727-bib-0078] Yu, G. , Wang, L.‐G. , Han, Y. & He, Q.‐Y. 2012 clusterProfiler: an R package for comparing biological themes among gene clusters. OMICS, 16:284–287. 22455463 10.1089/omi.2011.0118 PMC3339379

