# Supplementary material for: Differential Gene Expression Supports a Resource‐Intensive, Defensive Role for Colony Production in the Bloom‐Forming Haptophyte, Phaeocystis globosa
Source: J Eukaryot Microbiol. 2019 Mar 27;66(5):788–801. doi: 10.1111/jeu.12727 (PMC6766888; doi:10.1111/jeu.12727)
Supplement: Supplementary file 1 — Figure S1. Phylogenetic placement of Phaeocystis globosa strains CCMP1528 and CCMP2170. Figure S2. Correlations between External RNA Controls Consortium (ERCC) standard sequences’ initial concentrations and FPKM in colonial and solitary replicates. Figure S3. Percent of eukaryote and protist Benchmarking Universal Single Copy Orthologs (BUSCOs) complete, fragmented, or missing in Phaeocystis globosa CCMP1528 and Phaeocystis sp. CCMP2710 transcriptomes. Figure S4. Principal component analysis (PCA) and heatmap demonstrating gene expression patterns in colonial and solitary Phaeocystis globosa, including outlier sample C2. Figure S5. Relative abundance of bacterial 16S rRNA sequences present in the RNA extracted from colonial and solitary culture replicates. Table S1. Sequencing statistics for each sample before and after quality filtering. Table S2. Summary statistics for MMETSP Phaeocystis sp. CCMP2710 transcriptome and Phaeocystis globosa CCMP1528 transcriptome assembly. Table S3. Biological Process Gene Ontology (GO) terms overrepresented in significantly upregulated genes in colonial cultures: results from GOStats hypergeometric test. Table S4. Biological Process Gene Ontology (GO) terms overrepresented in significantly downregulated genes in colonial cultures: results from GOStats hypergeometric test. Table S5. KEGG pathways overrepresented in significantly upregulated genes in colonial cultures: results from ClusterProfiler enricher test. Table S6. KEGG pathways overrepresented in significantly downregulated genes in colonial cultures: results from ClusterProfiler enricher test. Table S7. KEGG pathways overrepresented in significantly upregulated genes in colonial cultures: results from edgeR kegga test. Table S8. KEGG pathways overrepresented in significantly downregulated genes in colonial cultures: results from edgeR kegga test. [file JEU-66-788-s001.pdf]

SUPPORTING INFORMATION

Differential Gene Expression Supports a Resource-Intensive, Defensive Role for Colony Production in the Bloom-Forming Haptophyte, *Phaeocystis globose* by Margaret Mars Brisbin and Satoshi Mitarai

Fig. S1. Phylogenetic placement of *Phaeocystis globosa* strains CCMP1528 and CCMP2170. Phylogenetic tree is built from a MUSCLE v3.8.31 alignment of *Phaeocystis* 18S rRNA gene sequences downloaded from GenBank (March 2018) using MrBayes v3.2.7 with six nucleotide substitution types (nst). Values associated with nodes are posterior probabilities as percent. The scale bar indicates 0.03 changes expected per site.

Fig. S2. Correlations between External RNA Controls Consortium (ERCC) standard sequences' initial concentrations and FPKM in colonial and solitary replicates. Quality-filtered sequencing reads for each culture replicate were mapped to ERCC standard sequences and read counts were determined with RSEM software. FPKM for sequences were log transformed and plotted against their log transformed initial concentrations in the standard mix within the R statistical environment. A linear model was fitted for each culture replicate with the lm function and the resulting regression line and R-squared are displayed on each plot. Plots were prepared with ggplot2.

Fig. S3. Percent of eukaryote and protist Benchmarking Universal Single Copy Orthologs (BUSCOs) complete, fragmented, or missing in *Phaeocystis globosa* CCMP1528 and *Phaeocystis sp.* CCMP2710 transcriptomes. BUSCO software was used to determine the percent of eukaryote and protist BUSCOs represented by complete single copies, complete but duplicated copies, copies that were fragmented or missing in the *Phaeocystis globosa* CCMP1528 transcriptome assembled for this study and the *Phaeocystis sp.* CCMP2710 transcriptome assembled for the Marine Microbial Eukaryote Transcriptome Sequencing Project (MMETSP). More eukaryote and protist BUSCOs were represented in the *Phaeocystis globosa* CCMP1528 transcriptome than the *Phaeocystis sp.* CCMP2710 transcriptome. Results plotted with R package ggplot2.

Fig. S4. Principal component analysis (PCA) and heatmap demonstrating gene expression patterns in colonial and solitary *Phaeocystis globosa*, including outlier sample C2. **A.** PCA performed on distances between samples derived from regularized log transformed counts. Results plotted with R package ggplot2. **B.** Heatmap including the 1,000 significantly differentially expressed genes with the lowest False Discovery Rate (FDR) adjusted p-values. Heatmap color represents differences from the mean regularized log transformed count for each contig in each sample. Gene expression in outlier sample C2 displays similarities to both colonial and solitary replicates. A group of 26 genes are uniquely upregulated in C2 and functional annotations for these were related to abiotic stress, defense (e.g. EF-hand_7, MACPF, Peroxidase), and cell growth (CRAL_TRIO, Actin). Results plotted with R package pheatmap.

Fig. S5. Relative abundance of bacterial 16S rRNA sequences present in the RNA extracted from colonial and solitary culture replicates. RNA extracts were reverse transcribed and then

processed following the Illumina guide for, “16S Metagenomic Sequencing Library Preparation,” before 300x300 bp paired-end amplicons were sequenced with an Illumina MiSeq using v3 chemistry. Reads were processed and annotated using the Qiime2 software and the SILVA ribosomal RNA reference database. Relative abundances of bacterial families in the RNA extracted from the colonial (C1-4) and solitary (S1-4) cultures were plotted with the Bioconductor package phyloseq. The Bray-Curtis distance between community compositions in samples was computed with phyloseq and distances between the two sample types were statistically significant when tested by PERMANOVA using the adonis function in the R package vegan ($p = 0.03$, $R^2 = 0.74$, 999 permutations).

Table S1. Sequencing statistics for each sample before and after quality filtering.

Table S2. Summary statistics for MMETSP *Phaeocystis* sp. CCMP2710 transcriptome and *Phaeocystis globosa* CCMP1528 transcriptome assembly.

Table S3. Biological Process Gene Ontology (GO) terms over-represented in significantly upregulated genes in colonial cultures: results from GOStats hypergeometric test.

Table S4. Biological Process Gene Ontology (GO) terms over-represented in significantly downregulated genes in colonial cultures: results from GOStats hypergeometric test.

Table S5. KEGG pathways over-represented in significantly upregulated genes in colonial cultures: results from ClusterProfiler enricher test.

Table S6. KEGG pathways over-represented in significantly downregulated genes in colonial cultures: results from ClusterProfiler enricher test.

Table S7. KEGG pathways over-represented in significantly upregulated genes in colonial cultures: results from edgeR kegga test.

Table S8. KEGG pathways over-represented in significantly downregulated genes in colonial cultures: results from edgeR kegga test.

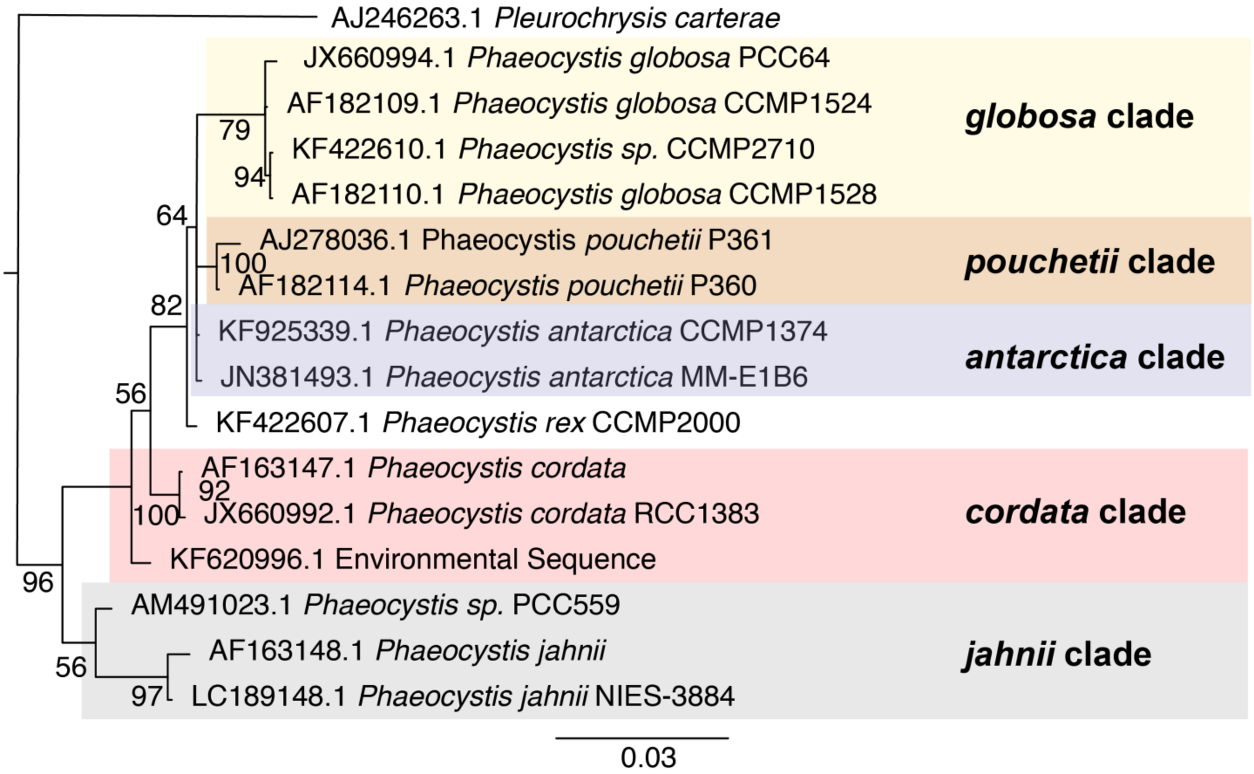

AJ246263.1 Pleurochrysis carterae
JX660994.1 Phaeocystis globosa PCC64
AF182109.1 Phaeocystis globosa CCMP1524
KF422610.1 Phaeocystis sp. CCMP2710
AF182110.1 Phaeocystis globosa CCMP1528
globosa clade
AJ278036.1 Phaeocystis pouchetii P361
AF182114.1 Phaeocystis pouchetii P360
pouchetii clade
KF925339.1 Phaeocystis antarctica CCMP1374
JN381493.1 Phaeocystis antarctica MM-E1B6
antarctica clade
KF422607.1 Phaeocystis rex CCMP2000
AF163147.1 Phaeocystis cordata
JX660992.1 Phaeocystis cordata RCC1383
KF620996.1 Environmental Sequence
cordata clade
AM491023.1 Phaeocystis sp. PCC559
AF163148.1 Phaeocystis jahnii
LC189148.1 Phaeocystis jahnii NIES-3884
jahnii clade
79
94
64
100
82
56
92
100
96
56
97
0.03

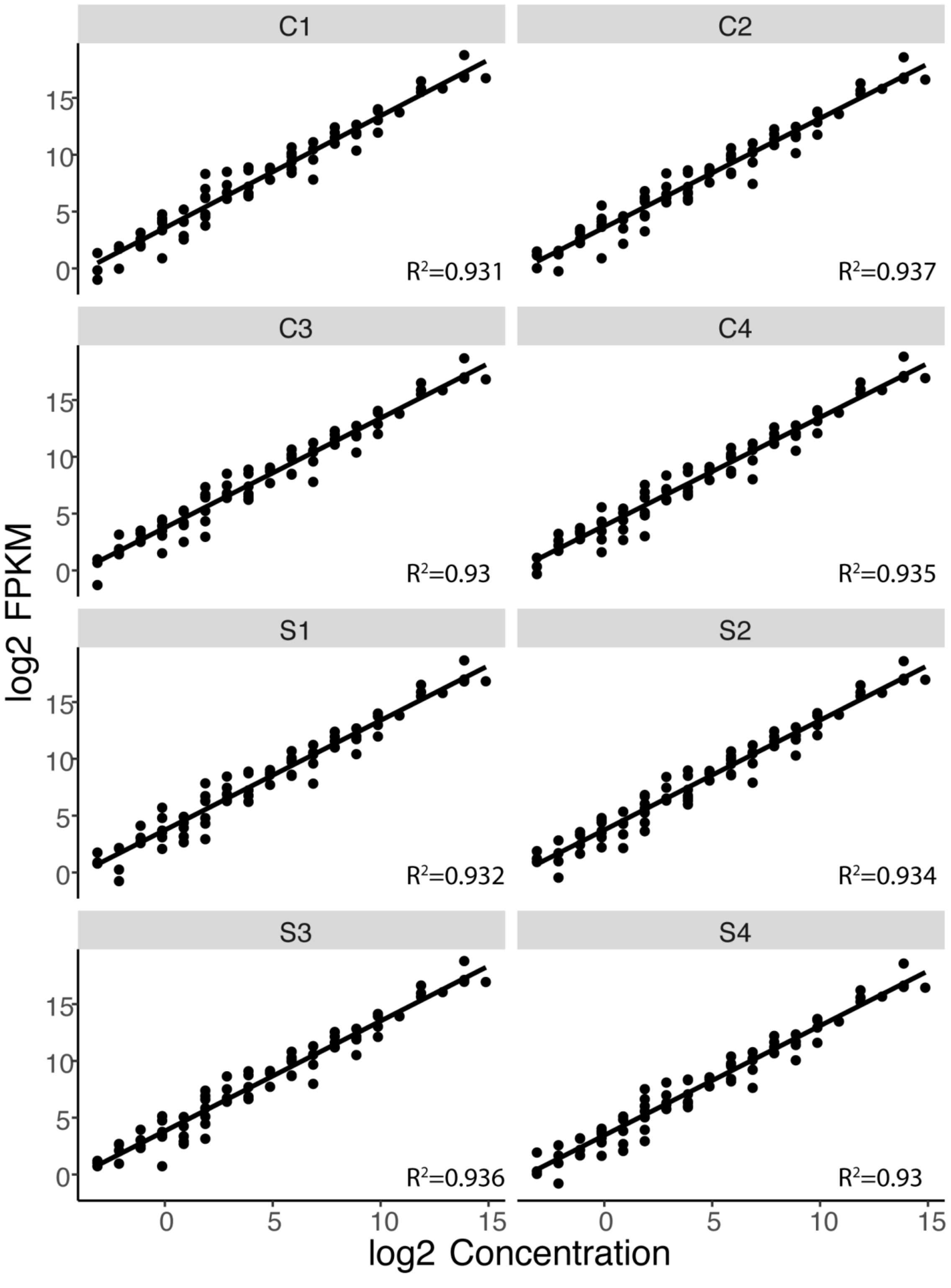
C1
C2
C3
C4
S1
S2
S3
S4
R²=0.931
R²=0.937
R²=0.93
R²=0.935
R²=0.932
R²=0.934
R²=0.936
R²=0.93
0
5
10
15
log2 FPKM
log2 Concentration

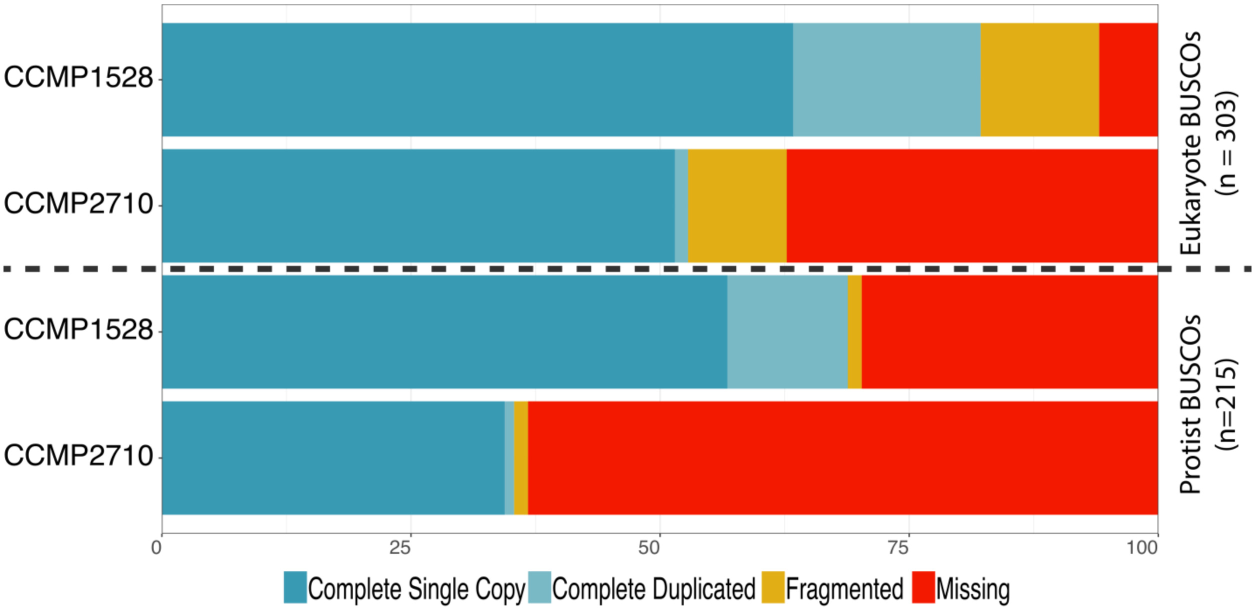
CCMP1528
CCMP2710
CCMP1528
CCMP2710
Eukaryote BUSCOs (n = 303)
Protist BUSCOs (n=215)
0
25
50
75
100
Complete Single Copy
Complete Duplicated
Fragmented
Missing

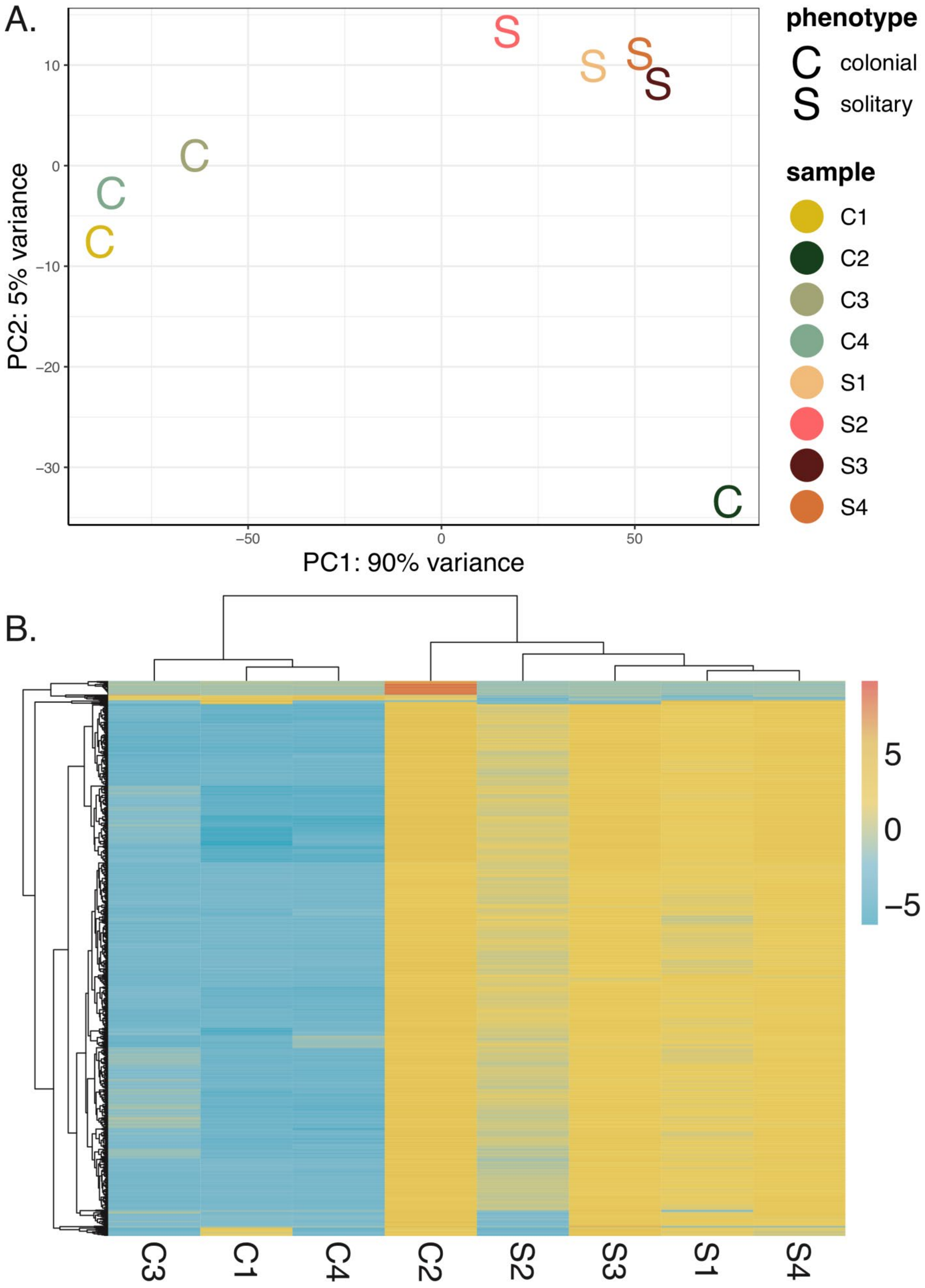
A.
PC2: 5% variance
PC1: 90% variance
10
0
−10
−20
−30
−50
0
50
phenotype
colonial
solitary
sample
C1
C2
C3
C4
S1
S2
S3
S4
B.
5
0
−5
C3
C1
C4
C2
S2
S3
S1
S4

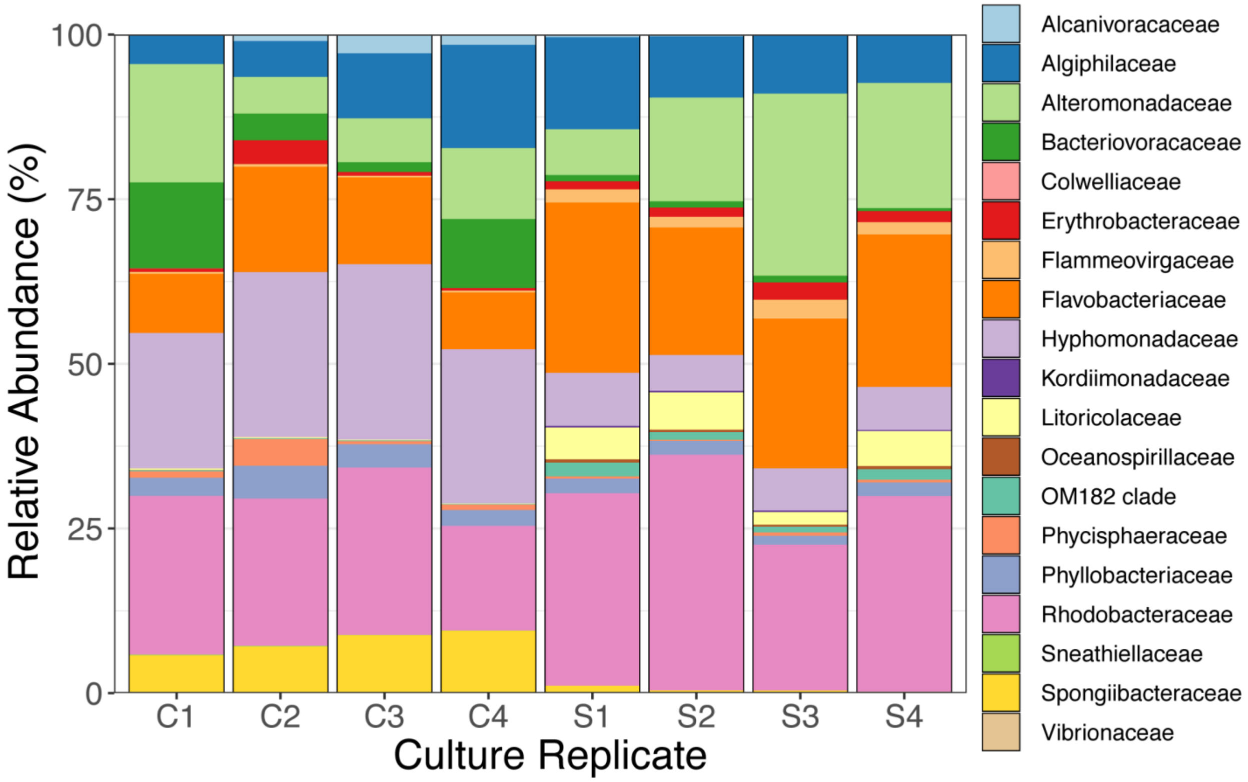

Relative Abundance (%)
100
75
50
25
0
C1
C2
C3
C4
S1
S2
S3
S4
Culture Replicate
Alcanivoracaceae
Algiphilaceae
Alteromonadaceae
Bacteriovoracaceae
Colwelliaceae
Erythrobacteraceae
Flammeovirgaceae
Flavobacteriaceae
Hyphomonadaceae
Kordiimonadaceae
Litoricolaceae
Oceanospirillaceae
OM182 clade
Phycisphaeraceae
Phyllobacteriaceae
Rhodobacteraceae
Sneathiellaceae
Spongiibacteraceae
Vibrionaceae

Table S1. Sequencing statistics for each sample before and after quality filtering.

	Total Sequence pairs	Base pairs	Quality Filtered	Base pairs
**C1**	159,410,644	48,013,094,646	140,028,684	41,561,197,264
**C2**	161,194,506	48,553,981,168	134,580,486	39,908,200,982
**C3**	163,861,064	49,355,447,786	142,755,477	42,292,222,468
**C4**	162,044,655	48,808,773,234	142,276,463	42,235,338,648
**S1**	288,990,302	87,047,243,376	250,244,322	68,490,645,624
**S2**	300,040,368	90,366,629,932	260,505,068	77,298,488,936
**S3**	383,465,686	115,483,974,368	341,859,220	101,634,303,704
**S4**	345,309,764	103,997,708,976	304,669,184	90,433,468,732

Table S2. Summary statistics for MMETSP *Phaeocystis* sp. CCMP2710 transcriptome and *Phaeocystis globosa* CCMP1528 transcriptome assembly.

	**MMETSP *Phaeocystis sp.* CCMP2710**	***Phaeocystis globosa* CCMP1528**
**# of contigs**	21,778	69,528
**Smallest contig (bp)**	150	201
**Largest contig (BP)**	9144	11817
**Mean contig size (BP)**	656	631
**Total n bases**	14,928,049	43,909,355
**Contigs mapping to other Transcriptome**	55%	18%

Table S3. Biological Process Gene Ontology (GO) terms over-represented in significantly upregulated genes in colonial cultures: results from GOStats hypergeometric test.

GO ID	Pvalue	OddsRatio	ExpCount	Count	Size	Term
**GO:0050794**	0.005818	2.584	5.33756	12	447	regulation of cellular process
**GO:0050789**	0.006811	2.527	5.44503	12	456	regulation of biological process
**GO:0065007**	0.011137	2.352	5.80326	12	486	biological regulation
**GO:0019321**	0.011940	Inf	0.01194	1	1	pentose metabolic process
**GO:0019646**	0.011940	Inf	0.01194	1	1	aerobic electron transport chain
**GO:0046373**	0.011940	Inf	0.01194	1	1	L-arabinose metabolic process
**GO:0019566**	0.011940	Inf	0.01194	1	1	arabinose metabolic process
**GO:0006793**	0.023889	1.911	9.60045	16	804	phosphorus metabolic process
**GO:0006796**	0.023889	1.911	9.60045	16	804	phosphate-containing compound metabolic process
**GO:0031323**	0.025822	3.218	1.68366	5	141	regulation of cellular metabolic process
**GO:0019222**	0.030218	3.078	1.75530	5	147	regulation of metabolic process
**GO:0007165**	0.031772	2.708	2.40011	6	201	signal transduction
**GO:0023052**	0.031772	2.708	2.40011	6	201	signaling
**GO:0007154**	0.033822	2.666	2.43593	6	204	cell communication
**GO:0006468**	0.034457	1.966	6.79435	12	569	protein phosphorylation
**GO:0042548**	0.035403	42.024	0.03582	1	3	regulation of photosynthesis, light reaction
**GO:0042549**	0.035403	42.024	0.03582	1	3	photosystem II stabilization
**GO:0010109**	0.035403	42.024	0.03582	1	3	regulation of photosynthesis
**GO:0043467**	0.035403	42.024	0.03582	1	3	regulation of generation of precursor metabolites

						and energy
**GO:0051716**	0.04945	2.244	3.36732	7	282	cellular response to stimulus

Table S4. Biological Process Gene Ontology (GO) terms over-represented in significantly downregulated genes in colonial cultures: results from GOStats hypergeometric test.

GO ID	Pvalue	OddsRatio	ExpCount	Count	Size	Term
**GO:0051641**	0.00000	2.277	38.51326	62	123	cellular localization
**GO:0051649**	0.00000	2.234	33.81652	54	108	establishment of localization in cell
**GO:0046907**	0.00000	2.191	33.5034	53	107	intracellular transport
**GO:0006886**	0.00038	2.086	29.1197	45	93	intracellular protein transport
**GO:0042886**	0.00065	1.898	35.3821	52	113	amide transport
**GO:0006508**	0.00069	1.424	124.620	154	398	proteolysis
**GO:0015031**	0.00076	1.892	34.7558	51	111	protein transport
**GO:0015833**	0.00076	1.892	34.7558	51	111	peptide transport
**GO:0045184**	0.00076	1.892	34.7558	51	111	establishment of protein localization
**GO:0070727**	0.00077	1.967	30.6853	46	98	cellular macromolecule localization
**GO:0034613**	0.00077	1.967	30.6853	46	98	cellular protein localization
**GO:0016192**	0.00084	1.867	35.6952	52	114	vesicle-mediated transport
**GO:0008104**	0.00137	1.807	36.3214	52	116	protein localization
**GO:0006413**	0.00471	3.009	8.14101	15	26	translational initiation
**GO:0006333**	0.00480	13.206	2.19181	6	7	chromatin assembly or disassembly
**GO:0033036**	0.00611	1.595	41.9575	56	134	macromolecule localization
**GO:0006325**	0.00757	2.758	8.45413	15	27	chromatin organization
**GO:0044085**	0.00859	1.594	38.2001	51	122	cellular component biogenesis
**GO:0070838**	0.0089	2.807	7.82789	14	25	divalent metal

	4					ion transport
**GO:0006334**	0.0133 0	10.998	1.87869	5	6	nucleosome assembly
**GO:0031497**	0.0133 0	10.998	1.87869	5	6	chromatin assembly
**GO:0051640**	0.0135 6	5.136	3.13115	7	10	organelle localization
**GO:0065004**	0.0135 6	5.136	3.13115	7	10	protein-DNA complex assembly
**GO:0007034**	0.0135 6	5.136	3.13115	7	10	vacuolar transport
**GO:0043933**	0.0157 6	1.726	22.8574	32	73	protein-containing complex subunit organization
**GO:0022607**	0.0188 7	1.647	25.6755	35	82	cellular component assembly
**GO:0046903**	0.0202 2	3.303	4.69673	9	15	secretion
**GO:0010033**	0.0202 2	3.303	4.69673	9	15	response to organic substance
**GO:0032940**	0.0202 2	3.303	4.69673	9	15	secretion by cell
**GO:0072511**	0.0206 7	2.374	8.45413	14	27	divalent inorganic cation transport
**GO:0006887**	0.0235 9	3.522	4.07050	8	13	exocytosis
**GO:0071824**	0.0273 0	3.85	3.44427	7	11	protein-DNA complex subunit organization
**GO:0019538**	0.0274 2	1.134	492.844	523	157 4	protein metabolic process
**GO:0071840**	0.0286 8	1.326	69.5117	83	222	cellular component organization or biogenesis
**GO:0065003**	0.0294 7	1.671	20.3525	28	65	protein-containing complex assembly
**GO:0015693**	0.0297	2.403	7.2016	12	23	magnesium ion

	0					transport
**GO:0009611**	0.03066	Inf	0.9393	3	3	response to wounding
**GO:0006904**	0.03115	4.399	2.8180	6	9	vesicle docking involved in exocytosis
**GO:0006323**	0.03115	4.399	2.8180	6	9	DNA packaging
**GO:0140056**	0.03115	4.399	2.8180	6	9	organelle localization by membrane tethering
**GO:0048278**	0.03115	4.399	2.8180	6	9	vesicle docking
**GO:0140029**	0.03115	4.399	2.8180	6	9	exocytic process
**GO:0022406**	0.03115	4.399	2.8180	6	9	membrane docking
**GO:0034728**	0.03455	5.497	2.1918	5	7	nucleosome organization
**GO:1901700**	0.03455	5.497	2.1918	5	7	response to oxygen-containing compound
**GO:0006487**	0.04018	2.934	4.3836	8	14	protein N-linked glycosylation
**GO:1901605**	0.04721	1.803	12.524	18	40	alpha-amino acid metabolic process
**GO:0007010**	0.04953	2.04	8.4541	13	27	cytoskeleton organization

Table S5. KEGG pathways over-represented in significantly upregulated genes in colonial cultures: results from ClusterProfiler enricher test.

ID	pvalue	qvalue	Count	Name
**ko04022**	0.00213369	0.07187163	3	cGMP-PKG signaling pathway

Table S6. KEGG pathways over-represented in significantly downregulated genes in colonial cultures: results from ClusterProfiler enricher test.

ID	pvalue	qvalue	Count	Name
**ko04142**	1.56E-05	0.00187389	44	Lysosome
**ko04016**	8.28E-05	0.00465393	17	MAPK signaling pathway - plant
**ko01521**	0.00011635	0.00465393	32	EGFR tyrosine kinase inhibitor resistance
**ko04140**	0.00029628	0.00888838	25	Autophagy - animal
**ko04152**	0.00829389	0.19905331	17	AMPK signaling pathway

Table S7. KEGG pathways over-represented in significantly upregulated genes in colonial cultures: results from edgeR kegga test.

Pathway	N	DE	p	Name
**ko04151**	47	3	0.016094	PI3K-Akt signaling pathway
**ko00601**	3	1	0.033879	Glycosphingolipid biosynthesis - lacto and neolacto series
**ko04022**	23	3	0.002133	cGMP-PKG signaling pathway
**ko04626**	24	2	0.030142	Plant-pathogen interaction
**ko04216**	3	1	0.033879	Ferroptosis
**ko04712**	4	2	0.0007586	Circadian rhythm - plant
**ko05203**	2	1	0.0227138	Viral carcinogenesis

Table S8. KEGG pathways over-represented in significantly downregulated genes in colonial cultures: results from edgeR kegga test.

Pathway	N	DE	p	Name
**ko04141**	138	63	0.04687	Protein processing in endoplasmic reticulum
**ko04014**	50	27	0.01757	Ras signaling pathway
**ko00190**	98	47	0.03260	Oxidative phosphorylation
**ko04142**	69	44	0.00000	Lysosome
**ko04016**	21	17	0.00000	MAPK signaling pathway - plant
**ko01521**	49	32	0.00011	EGFR tyrosine kinase inhibitor resistance
**ko04152**	27	17	0.00829	AMPK signaling pathway
**ko00100**	23	14	0.02393	Steroid biosynthesis
**ko00600**	28	16	0.03379	Sphingolipid metabolism
**ko04140**	37	25	0.00029	Autophagy - animal
**ko04742**	8	7	0.00650	Taste transduction
**ko00071**	28	16	0.03379	Fatty acid degradation
